# TREM2 Impedes Recovery After Spinal Cord Injury by Regulating Microglial Lysosomal Membrane Permeabilization‐Mediated Autophagy

**DOI:** 10.1111/cpr.70047

**Published:** 2025-05-04

**Authors:** Tianlun Zhao, Jiawei Di, Yu Kang, Haojie Zhang, Senyu Yao, Bin Liu, Limin Rong

**Affiliations:** ^1^ Department of Spine Surgery The Third Affiliated Hospital of Sun Yat‐Sen University Guangzhou China; ^2^ Guangdong Provincial Center for Engineering and Technology Research of Minimally Invasive Spine Surgery Guangzhou China

**Keywords:** autophagy, lysosomal membrane permeabilization, microglia, spinal cord injury, TREM2

## Abstract

Microglia, considered as the main immune responder, play an important role in regulating neuroinflammation in central nervous system (CNS) disorders. Our previous work found that TREM2 is highly expressed in microglia and is related to their functional state. However, the specific role of TREM2 in spinal cord injury has not yet been explored. To further investigate the potential mechanism of TREM2, we performed single‐cell sequencing on wild‐type (Wt) and *Trem2* ^*−/−*^ mice before and after spinal cord injury. Compared to Wt mice, the lysosome, autophagy and membrane‐related pathways are more strongly activated in *Trem2* ^*−/−*^ mice, suggesting that TREM2 may exert its effects by influencing lysosomal membranes and autophagy. Mechanistically, we demonstrated that the knockout of *Trem2* can reduce the nuclear translocation of TFEB by decreasing the phosphorylation of Syk. Furthermore, we validated that in vitro and in vivo silencing *Trem2* can promote autophagy by repairing lysosomal membrane permeabilization. Through immunofluorescence, 3D gait analysis, motor evoked potential experiments, H&E staining and Masson staining, we demonstrated that the increased level of autophagy can rescue more microglia in vivo and promote both functional and histological recovery of spinal cord injury. Collectively, these results not only suggest that microglial lysosomal autophagy is regulated in a TREM2‐dependent LMP manner, but also, more importantly, they provide a promising clinical translation strategy based on gene therapy for lysosome‐related central nervous system disorders.

## Graphical abstract

TREM2 exacerbates spinal cord injury by inhibiting TFEB nuclear translocation, aggravating lysosomal membrane permeabilisation and consequently suppressing autophagy.

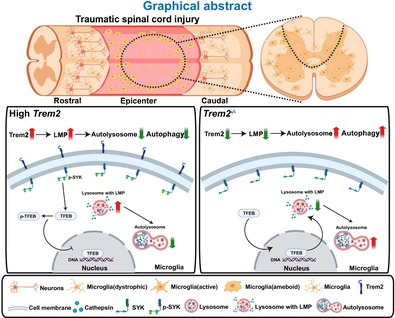

## 1 Introduction

Spinal cord injury (SCI) is a serious neurological disorder that frequently results in significant disability and substantial economic burdens [1]. As of 2019, global cases of SCI are approximately 9 million, marking a 52.7% increase since 1990, with hundreds of thousands of new cases each year [2]. Despite such prevalence, highly effective treatments are still exceedingly limited. The pathophysiology of SCI is divided into primary and secondary injuries [3]. The primary injury involves direct mechanical damage, usually irreversible. Following the primary injury, secondary injury occurs, during which there is extensive death of microglia, followed by local proliferation to replenish their numbers. Previous studies have shown that depletion of microglia before injury significantly worsens the damage; thus, protecting the number of these cells has become one of the therapeutic strategies [4].

Triggering Receptor Expressed on Myeloid Cells 2 (TREM2) is considered a major immune signalling centre in central nervous system (CNS) disorders, highly expressed in microglia and regulating various functions [5]. For example, TREM2 is essential for the full activation of the microglial phenotype associated with neurodegenerative diseases—known as disease‐associated microglia (DAM) [5, 6]. However, the role of TREM2 in spinal cord injury has been rarely reported. Our analysis of existing single‐cell sequencing data shows that *Trem2* is closely related to the lysosomal pathway [7]. Therefore, we will focus our research on this area.

Lysosomal membrane permeabilization (LMP) is the disruption of the integrity of the lysosomal membrane, resulting in the leakage of lysosomal contents such as hydrolases into the cytoplasm [8, 9, 10]. This leakage can lead to reduced lysosomal activity and cellular damage. Inhibiting LMP is crucial for maintaining cellular functions. In spinal cord injury research, there is extensive study on neuronal LMP (lysosomal membrane permeability), but relatively less focus on LMP in microglia. However, the literature proves that LMP also plays an important role in microglia [11].

Autophagy is a lysosome‐dependent catabolic pathway that degrades cytoplasmic proteins and organelles to provide energy for cellular activities, thereby maintaining cellular homeostasis and enhancing cell survival [12]. The autophagic process includes the formation of phagophores, their development into autophagosomes, fusion with lysosomes to form autolysosomes and the subsequent degradation of these autolysosomes [13]. Therefore, when lysosomal activity decreases, it weakens the autophagic flux, leading to reduced autophagy and ultimately resulting in an increased mortality rate of microglia.

In this study, we bred a *Trem2* knockout mouse model to explore the role of the *Trem2* in spinal cord injury (SCI) and its potential mechanisms. Through experiments such as motor evoked potential (MEP), BMS score and BehaviorAtlas 3D Gait Analysis [14], we found that *Trem2* knockout mice exhibited improved functional recovery after SCI. Further single‐cell sequencing analysis revealed that this improvement may be associated with enhanced lysosomal membrane integrity, lysosomal activity and autophagic processes. To further explore the underlying mechanism, we employed techniques such as transmission electron microscopy, immunofluorescence and Western blot, and performed both positive and negative validation using the lysosomal membrane permeabilization inducer *N*‐dodecylimidazole (NDI) [15]. The results indicate that the knockout of *Trem2* can enhance lysosomal biogenesis by promoting the nuclear translocation of TFEB (transcription factor EB), repair lysosomal membrane permeability, improve lysosomal activity and increase autophagic activity.

This study deepens our understanding of TREM2 and its regulation of lysosomal membrane permeability changes, providing new potential therapeutic targets and pathophysiological processes for intervention in the treatment of spinal cord injury.

## 2 Materials and Methods

### 2.1 Mice

*Trem2* ^*−/−*^ mice (The Jackson Laboratory, #027197), Cx3cr1^CreER+/−^ mice (The Jackson Laboratory, #021160) and Rosa26^TdTomato+/−^ mice (The Jackson Laboratory, #007914) were on the C57BL/6 genetic background. Cx3cr1^CreER^: R26‐TdTomato mice were generated by crossing Cx3cr1^CreER+/−^ with Rosa26^TdTomato+/+^ mice. Comprehensive details concerning the transgenic mice are presented in Table S1. Cx3cr1^CreER^: Rosa26^TdTomato+/+^ mice allow microglia in their bodies to emit red fluorescence, making them commonly used for labelling microglia. Mice were group‐housed in the virus/antigen‐free Animal Core Facility of Sun Yat‐sen University (#SYXK 2023‐0112), with a 12–12 h light–dark cycle with free access to food and water. Animal care and experiments were performed according to the protocol approved by Sun Yat‐sen University. Adult (8–12‐weeks) female mice were used in this study. Healthy mice were randomly assigned to experimental groups. This study was conducted using a blinded approach. The specific procedures for blinding were as follows: First, mice were randomly assigned to either the sham surgery group or the injury group. Second, each mouse was assigned a unique alphanumeric code (e.g., A01, B02). Investigators performed surgeries, postoperative behavioural tests and sample collection based solely on these codes. Finally, after data analysis was completed, an independent researcher decoded the group assignments and performed statistical comparisons between groups [16].

### 2.2 Contusive Spinal Cord Injury (SCI) Surgery

Anaesthetised mice received a laminectomy at thoracic vertebra 9 (T9), then a moderate (70 kD) contusion on the exposed spinal cord using the Infinite Horizon Impactor (Precision Systems and Instrumentation LLC) with an 800 μm diameter tip. All mice that survived the surgery and had contusions with recorded impact forces from 70kD to 80kD and displacements from 500 to 700 μm were included in the study. After surgery, mice received antibiotics and analgesics subcutaneously once daily for the first week. Bladder expressions were conducted twice daily for the first week, then once daily for the rest of the study [7]. We used 8–12‐week‐old female C57BL/6 mice to establish the contusive SCI model. A laminectomy (sham‐operated) group without SCI damage was used as the control.

### 2.3 Data Analysis of scRNA‐Seq

By searching for “spinal cord injury” in the GEO Database, we retrieved the dataset GSE162610 (submitted by James S. Choi in 2020) (10.1084/jem.20210040), which includes uninjured and injured spinal cord samples at 1 dpi, 3 dpi and 7 dpi from wild‐type mice. We analysed the uploaded scRNA‐seq data from *Trem2* ^*−/−*^ mice (10.1016/j.celrep.2023.112629). Gene expression counts were normalised to counts per 10,000 and log‐transformed, and the top 3000 variable genes were identified using the “vst” method in Seurat (v4.4.0) (10.1016/j.cell.2021.04.048). Gene expression values were then scaled and centred, and the resulting data were input into Harmony, which was run using 30 principal components. The integrated Harmony embeddings were subsequently used for *k*‐nearest neighbour graph construction and Leiden clustering, following the default Seurat workflow (10.1038/s41592‐019‐0619‐0). We visualised the distribution of cell types, experimental groups, and marker gene expression across the entire dataset and original cell types using t‐SNE embeddings. Differentially expressed genes for the subpopulations of interest were identified using the FindMarkers function. Based on the identified differentially expressed genes, we further conducted functional annotation and Gene Set Enrichment Analysis (GSEA), focusing on GO Cellular Component and KEGG pathways (0.1038/75556) (10.1093/nar/28.1.27). Visualisation of enriched pathways was performed using clusterProfiler and ggplot2 to plot enrichment scores and pathway significance (10.1038/s41596‐024‐01020‐z). Besides, the Spearman correlation was calculated between *Trem2* and other genes across cells, with statistical significance determined using a *p*‐value threshold of < 0.05. The correlation was visualised using a scatter plot, with a linear regression line plotted to illustrate the strength and direction of the correlation [17].

### 2.4 Single‐Cell RNA Sequencing (scRNA‐Seq)

Single‐cell RNA sequencing was performed on myeloid cell enriched suspensions obtained from the spinal cord of naive, SCI (acute) mice. The spinal cord was enzymatically dissociated into single‐cell suspensions using a papain‐based Neural Dissociation Kit (Miltenyi, #130‐092‐628). After myelin removal, myeloid cells were enriched by positive selection with CD11b Microbeads (Miltenyi, #130‐049‐601) according to the manufacturer's protocol. For high‐throughput sample processing and batch effect elimination, single‐cell suspensions from three samples were pooled using the BD Single‐cell Multiplexing Kit (BD, #633793). Library preparation was performed using the BD Rhapsody system according to the manufacturer's protocol. Sequencing was conducted with an Illumina Novaseq 6000 PE150 instrument. The GEO accession numbers are GSE162610 and GSE198852.

### 2.5 Immunofluorescence

The T9 spinal cord segments of anaesthetised mice were completely removed after heart perfusion with saline and 4% paraformaldehyde. The spinal cord tissue was then embedded in OCT after 3 h of 4% paraformaldehyde fixation and 2 days of gradient sucrose dehydration. The embedded tissue was cut into 20 μm‐thick sections vertically or transversely with a microtome. For tissue immunofluorescence staining, OCT on the tissue surface was rinsed off with PBS, sections were permeabilized in 0.4% Triton X 100 (solved in PBS) for 2 h and blocked with 0.5% BSA (solved in PBS) for 1 h, followed by treatment with primary antibodies overnight at 4°C (Gal3(1:500), CTSL(1:500), Lamp1(1:500), P2Y12(1:500), Iba1(1:1000), LC3B(1:500), P62(1:500), TREM2(1;500), GFAP(1:1000), MAP2(1:500)). Next, secondary antibodies were incubated at room temperature for 2 h. Finally, sections were rinsed in water before mounting with ProLong Gold Antifade Reagent with DAPI (P36971, Thermo, USA). In both coronal and transverse sections, we used GFAP to define the injury boundaries of the spinal cord and focused on capturing images from the area with the most severe damage. We define the area inside the injury boundary determined by GFAP as the “damage epicentre,” the region extending 250 μm outward from the injury boundary as the “epicentre boundary,” and the area beyond 250 μm as “beyond the epicentre.” Images were acquired using LSM800 confocal microscope (Zeiss) or Dragonfly CR‐DFLY‐2022540 (Leica). The antibodies used in the experiment are listed in Table S2.

### 2.6 Morphological Quantification of Microglia

For in vivo analysis, 20× confocal microglia pictures were taken from 20 μm thick sections staining for P2RY12. Images were processed with Leica LAS X and ImageJ software and conducted FracLac analysis (https://imagej.nih.gov/ij/plugins/fraclac/FLHelp/Introduction.htm). Briefly, 10 confocal pictures were collected from each animal for microglial morphology analysis. About 20 microglia cells were randomly selected from those confocal pictures in each animal. At least 50 microglia cells were analysed in each genotype. Images were then converted to 8‐bit grayscale before being processed to binary and outline images using ImageJ installed with AnalyzeSkeleton (2D/3D) plugin [18]. Detailed morphological information was provided using plugin FracLac.

### 2.7 Protein Extraction and Western Blotting

Seven days after spinal cord injury, the mice were euthanized, and the spinal cord tissue was collected. Samples of spinal cord tissue (epicentre ±0.5 cm) cultured cell samples were lysed by ultrasonication in RIPA buffer with protease and phosphatase inhibitors (P1005, Beyotime, Shanghai, China). Protein concentrations were measured using a BCA kit (23,225, Thermo, USA), and sample concentrations were adjusted accordingly. The samples were then denatured in loading buffer, electrophoresed on 8%–12% SDS‐PAGE gels, and subsequently transferred to PVDF membranes. Membranes were blocked in 5% BSA and treated with primary antibodies (Lamp1(1:1000), CTSL(1;1000), CTSD(1:1000), CTSC(1;1000), GAPDH(1:5000), Beclin‐1(1;1000), LC3(1:1000), P62(1:2000), Syk(1:1000), p‐Syk(1:1000), TFEB(1:1000), Histone H3(1:1000)) before probing with secondary antibodies (1 h, room temperature). Details of the antibodies are given in Table S2. Signal intensities were detected with ECL and quantified by ImageJ software (v1.53a, NIH, USA) [19].

### 2.8 Motor Evoked Potential (MEP)

This was used to assess nervous system function in the hindlimbs on day 35 following SCI, using the BL‐420A/F Data Acquisition Analysis System (TECHMAN SOFT). Electrodes were placed in the motor cortices and used to stimulate the brain, with the recording electrode positioned on the contralateral sciatic nerve. Motor neuron function was assessed by the amplitude of the first evoked peak.

### 2.9 Basso Mouse Scale (BMS) Score

The motor function of mice was assessed with BMS score after injury; each mouse was pretrained and individually placed in an open field. All mice were observed by two independent investigators blinded to the treatment groups on days 1, 3, 7, 14, 21, 28 and 35 after the injury. The BMS scale ranges from 0 (no ankle movement) to 9 (complete functional recovery) and rates locomotion based on hind limb joint movements, trunk position and stability, stepping coordination, paw placement, toe clearance and tail position.

### 2.10 BehaviorAtlas 3D Gait Analysis

3D gait data were collected using the BehaviorAtlas 3D‐AI Mouse Motor Function Capture system (V1.0.1, Shenzhen Bayone BioTech Co.), and analysed with the corresponding Motor Function Analyser software. The experiment included camera calibration, data collection and analysis. Calibration was ensured by capturing checkerboard images using a multi‐camera system for accurate alignment. Mice walked on a treadmill at 4 cm/s, with data captured from four synchronised views at 60 fps for 30 s. In the analysis phase, 17 key points were reconstructed in 3D, generating a 3D coordinate matrix and extracting gait parameters (such as gait cycle, stance/swing phase duration and kinematic parameters). The results were visualised through gait trajectory plots and other visualisation methods [14].

### 2.11 Subcellular Fractionation and Preparation of Lysosome‐Rich Fractions

After euthanising the mice, the spinal cord tissue was extracted, washed with cold PBS, and chopped using surgical scissors. Following the manufacturer's instructions (Lysosome Enrichment Kit for Tissue and Cultured Cells; Thermo Fisher Scientific, 89,839), the tissue was homogenised for 45 s at 8000 rpm using a Polytron Tissue Tearer in Reagent A. Reagent B was then added, and the homogenate was centrifuged to remove cell debris. The resulting supernatant was layered onto a discontinuous gradient of OptiPrep cell separation medium and subjected to differential centrifugation to separate and enrich lysosomes [20, 21].

### 2.12 Isolation and Enrichment of Nuclear Protein

Nuclear protein extraction was performed using a nuclear and cytoplasmic extraction kit (Thermo Scientific, 78,835). Cells were washed with PBS, lysed with cytoplasmic extraction buffers, and centrifuged to separate the cytoplasmic and nuclear fractions. The nuclear pellet was incubated with nuclear extraction buffer on ice for 40 min, followed by centrifugation to collect the supernatant containing nuclear proteins. Protein concentration was determined using the BCA assay, and samples were stored at −80°C for further use.

### 2.13 RNA Isolation and Real‐Time Quantitative PCR

Total RNA was extracted from spinal cord specimens with an E.Z.N.A. Total RNA Kit I (R6834‐02 Omega, USA) and reverse‐transcribed to cDNA with an Evo M‐MLV kit (AG11705 Accurate Biology China) for qPCR. Amplification of 20 μL reaction volumes was performed using PowerUp TM SYBR TM Green Master Mix (A25742; Thermo Fisher) on a QuantS system (Applied Biosystems). Three independent runs with duplicates were used. Gene expression was normalised to that of GAPDH using the 2^−△CT^ method [22]. The qPCR primers are shown in Table S3.

### 2.14 Transmission Electron Microscopy (TEM)

The mouse hearts were perfused with saline and 2.5% glutaraldehyde. Spinal cord tissues were harvested and placed in 2.5% glutaraldehyde, fixed overnight at 4°C. After fixation, the tissues were dehydrated and embedded, then sectioned into 70 nm slices, and examined and imaged under a transmission electron microscope (TEM) (HT7800/HT7700, Hitachi, Japan) at high contrast mode, with an acceleration voltage of 80 kV and a magnification of 8.0k [23].

### 2.15 *N*‐Dodecylimidazole (NDI) Treatment of Animals and Cells

For animal treatment: Mice were orally gavaged with 300 μL of NDI (HY‐138540, MCE, USA) solution (100 mg/kg/day) in saline after spinal cord injury surgery.

For cell treatment: NDI was added to the cell culture medium to achieve a final concentration of 40 μM [24].

### 2.16 Haematoxylin and Eosin (H&E) Staining

Thirty‐five days after the surgery, the mice were deeply anaesthetised and transcardially perfused, first with saline and then with 4% (w/v) paraformaldehyde (PFA). After embedding in OCT compound, the tissue was sectioned into 20 μm slices using a cryostat. The sections were stained with haematoxylin for 3–5 min to stain the cell nuclei, followed by rinsing with tap water, and then stained with eosin for 1–2 min to stain the cytoplasm. After staining, the sections were rinsed with water to remove excess dye, and then mounted using glycerol. Finally, the prepared slides were examined under a light microscope [25].

### 2.17 Franz Nissl Staining

After transcardial perfusion with saline and 4% (w/v) PFA, the spinal cord tissue was extracted and embedded. OCT‐embedded spinal tissues were cut longitudinally into 20 μm sections and stained with cresyl violet. After staining, the sections were rinsed with water to remove excess dye, and then mounted with glycerol or another suitable mounting medium. The Nissl bodies in the sections were observed and imaged under a microscope (Nikon) [26].

### 2.18 Masson Staining

Spinal cord tissues embedded in OCT were sectioned longitudinally into 20 μm slices and briefly rehydrated. Staining commenced with Weigert's iron haematoxylin for nuclei (5 min), followed by Biebrich Scarlet‐Acid Fuchsin for muscle fibres and cellular structures (5 min). Excess red stain was removed with a phosphomolybdic/phosphotungstic acid solution (5–10 min), and collagen was stained with aniline blue or light green solution (5 min). Sections were differentiated in 1% acetic acid, dehydrated through an ascending alcohol series, cleared in xylene, and mounted with aqueous glycerol jelly. Observations were conducted using a light microscope.

### 2.19 Enzyme‐Linked Immunosorbent Assay (ELISA)

The activity of CTSD (EIAab, E1280m) was determined with ELISA kits following the manufacturers' instructions. The optical density of the samples was measured using a microplate reader at 550 nm with a correction wavelength of 450 nm for quantification of CTSD.

### 2.20 Statistics and Reproducibility

All experiments were conducted with at least three biological replicates, and the results showed successful reproducibility. All data are reported as the mean ± standard error of the mean (SEM) from at least three independent experiments. Sample sizes are presented in the figure legends. Statistical analysis between two groups was performed using an unpaired *t*‐test. Statistical analysis between multiple groups was performed using one‐way ANOVA with multiple comparison tests. All data were analysed using GraphPad Software. A two‐sided *p*‐value < 0.05 was considered statistically significant. The level of significance was defined as *p < 0.05, **p < 0.01, ***p < 0.001 and ****p < 0.0001.

## 3 Result

### 3.1 Changes in Lysosomal Function After Spinal Cord Injury May Be Related to the Number, Morphology and Cluster of Microglia

**FIGURE 1:**
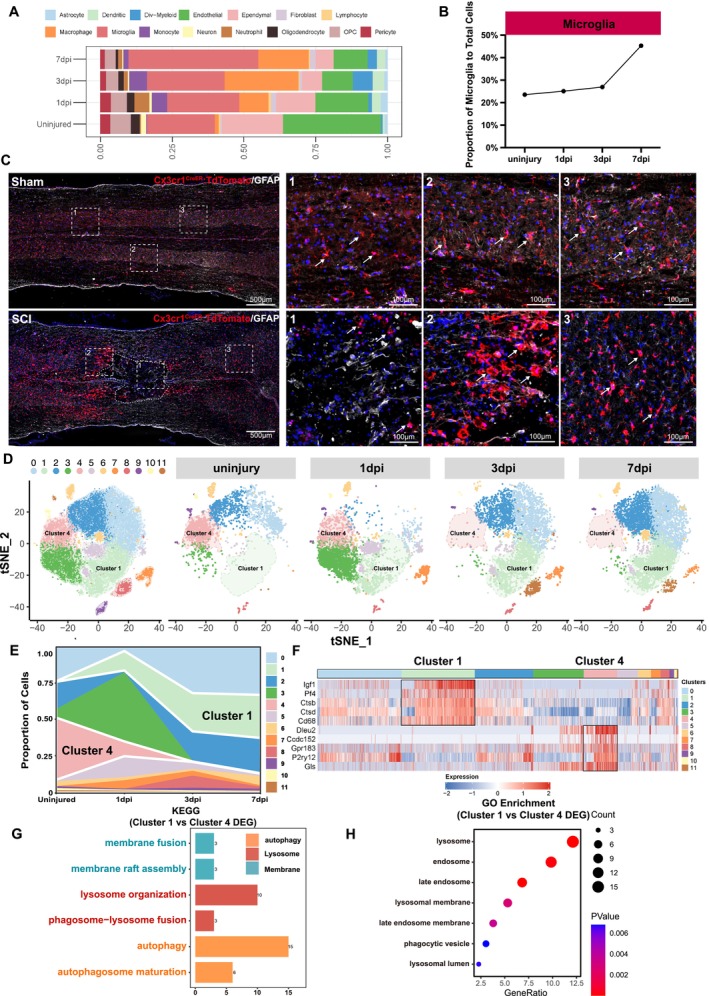
Microglia undergo significant changes in number, morphology, and function after spinal cord injury. (A) Proportional change in cell type composition over time post‐injury. The bar chart illustrates the relative proportions of different cell types at four time points: Uninjured, 1 dpi, 3 dpi and 7 dpi, based on single‐cell RNA‐seq analysis. (B) Line graph shows the changes in the proportion of microglia before and after spinal cord injury. (C) Representative immunofluorescence images of coronal frozen sections from the spinal cord, sham and 7 days post‐injury. (D) Re‐clustering of microglia at different time points following injury. t‐SNE plots show the re‐clustering of microglia populations at four distinct time points. Each colour represents different microglial subtypes, with clusters 1 and 4 highlighted in green and pink, respectively, to indicate key shifts in microglial states following injury. These changes suggest distinct functional roles for different microglial subpopulations in the response to injury over time. (E) Representative images illustrating the proportional changes of different microglial subtypes at various time points before and after spinal cord injury. (F) Heatmap of representative key genes of cluster 1 and cluster 4. Colour represents Z score expression level. Red, upregulated genes; blue, downregulated genes. (G) Pathway Functional Enrichment Analysis of Differentially Expressed Genes (DEG). The bar graph illustrates the pathways significantly regulated by differentially expressed genes in the two most distinct groups (cluster 1 vs. cluster 4) from (E). Orange represents autophagy‐related pathways, red indicates lysosomal‐related pathways, and blue denotes membrane‐related pathways. The *x*‐axis represents the number of differentially expressed genes enriched in the regulation of each pathway. (H) Cellular Component Distribution and Significance Analysis. Dot graph illustrating the quantitative distribution of differentially expressed genes across various cellular components at 2 clusters (cluster 1 vs. cluster 4). The *x*‐axis represents the Gene ratio of each component, whilst the *y*‐axis delineates the specific cellular components. The size of the circles represents the number of enriched genes, whilst the colour indicates the *p*‐value.

Traumatic spinal cord injury significantly alters the proportion of cells at the injury site. Utilising existing single‐cell sequencing data, we analysed t‐SNE plots of cell populations from groups at various stages: sham surgery, 1‐day post‐injury, 3 days post‐injury and 7 days post‐injury (Figure 1a) [7]. The number of microglia had increased to become the dominant cell type at the site of injury by the seventh day post‐injury, significantly (Figure 1a). The line graph illustrates the changes in the proportion of microglia, indicating that their proportion peaks 7 days after spinal cord injury (Figure 1b). This suggests that microglia play a crucial role in the response to spinal cord injury.

To further explore the changes in microglia, we bred a microglia‐specific fluorescent reporter mouse and performed spinal cord injury surgery [27]. Tissue samples were collected on the seventh day post‐injury and subjected to immunofluorescence staining (Figure 1c). The results demonstrated substantial differences in microglia density and morphology compared to the sham group. At the injury's core, microglia were sparse and displayed irregular shapes, indicative of significant cell death. In contrast, at the periphery of the injury, there was a pronounced increase in microglia density, and the cells were notably larger in size. In areas distant from the injury centre, the density and morphology of microglia resembled those of the sham group.

We hypothesise that microglia at the site of spinal cord injury may be divided into several groups. To explore this, we extracted microglia from existing single‐cell sequencing data and analysed their t‐SNE plots at different stages. We categorised the microglia into 11 distinct clusters (Figure 1d). We plotted a line graph of cell proportions, and the results showed significant changes in cluster 1 and 4 before and after spinal cord injury: in the sham surgery group, cluster 4 microglia were predominantly expressed; by contrast, 7 days post spinal cord injury, cluster 1 microglia were the main type expressed (Figure 1e). We further generated a heatmap to illustrate the specificity of cluster 1 and cluster 4. The results revealed that cluster 1 predominantly expresses *Igf1*, *Pf4*, *Ctsb*, *Ctsd* and *Cd68*, whilst cluster 4 primarily expresses *Dleu2*, *Ccdc152*, *Gpr183*, *P2ry12* and *Gls* (Figure 1f). We further validated CTSD, CD68, P2Y12 and GLS1 through immunofluorescence experiments. The results showed that in the uninjured state, the markers of cluster 1, *Ctsd* and *Cd68*, were scarcely expressed. However, on the 7th day after spinal cord injury, their expression levels were significantly upregulated. As for the markers of cluster 4, *P2ry12* and *Gls*, they were highly expressed when there was no injury, but their expression levels decreased significantly on the 7th day after spinal cord injury (Figure 1b,c). This result clearly indicates that cluster 4 is mainly expressed in the uninjured state, whilst cluster 1 is highly expressed after injury. This important finding provides crucial clues for further exploration of the functional heterogeneity of microglia.

We performed differential gene analysis between cluster 1 and 4 microglia. KEGG analysis revealed that, compared to cluster 4, cluster 1 microglia exhibited significantly enhanced activity in lysosome, autophagy and membrane‐related pathways (Figure 1g). We also conducted GO analysis and found that these differential genes were primarily expressed in lysosomes, endosomes, and their associated membrane structures. These findings highlight significant molecular and biological differences between the different microglial populations (Figure 1h). They also suggest that autophagy and lysosome‐related pathways undergo significant changes after spinal cord injury, playing a crucial role in the repair mechanisms. This insight provides important clues for advancing spinal cord injury research and therapeutic strategies.

### 3.2 *Trem2* Expression Rises After Spinal Cord Injury and Relates to Lysosomal Membrane Permeabilisation Genes

**FIGURE 2:**
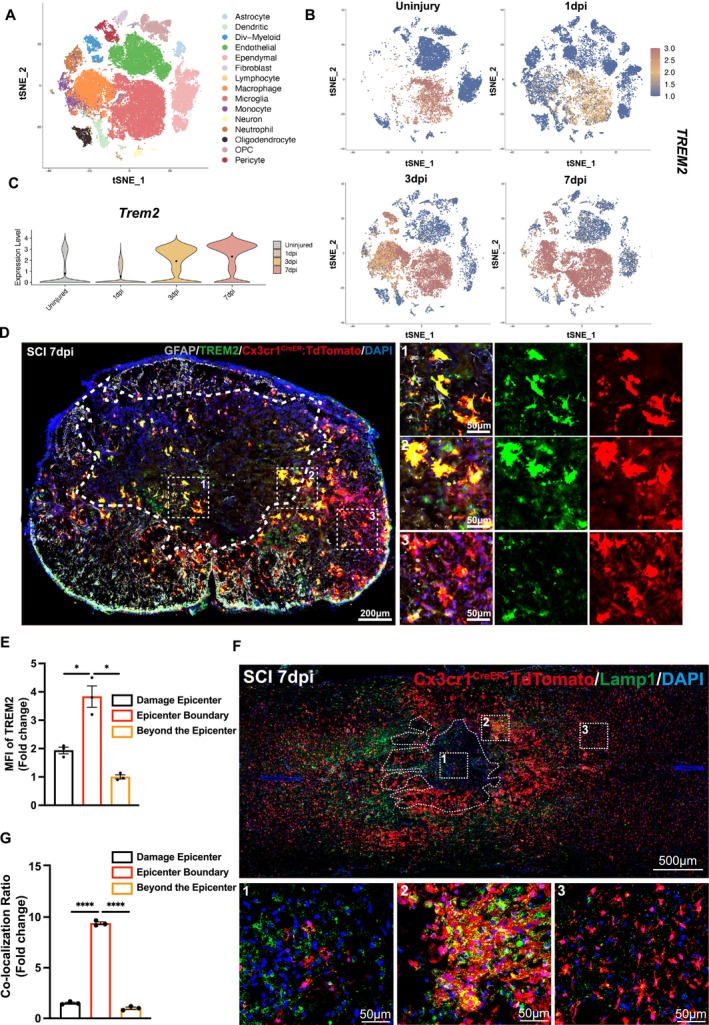
TREM2 and Lamp1 are primarily expressed in microglia at the boundary of the injury site. (A) Reclustered tSNE plots depicting WT cell populations. (B) tSNE plots showing expression of *Trem2* at different time points before and after SCI. (C) Violin plots showing the expression levels of TREM2 in the uninjured group and in the groups 1 day, 3 days and 7 days after injury. (D) Representative immunofluorescence images of TREM2 in Cx3cr1^CreER^:R26‐TdTomato mice at 7 days after SCI. Scale bar: 200 μm. (E) Quantify the average fluorescence intensity of TREM2 in different injury areas according to graph D. We used three samples for this experiment. For each sample, three regions were randomly selected from the “damage epicentre,” “epicentre boundary,” and “beyond the epicentre” for quantification, and the average values were calculated. Ordinary one‐way ANOVA. (F) Representative immunofluorescence images of Lamp1 in Cx3cr1^Cre^:R26‐TdTomato mice at 7 days after SCI. Scale bar: 500 μm. (G) Comparison of microglia and Lamp1 co‐localization area in different regions of spinal cord injury. We used three samples for this experiment. For each sample, three regions were randomly selected from the “damage epicentre,” “epicentre boundary,” and “beyond the epicentre” for quantification, and the average values were calculated. Ordinary one‐way ANOVA. All data are mean ± SEM; Error bars represent SEMs; *p < 0.05, **p < 0.01, ***p < 0.001, ****p < 0.0001, ns > 0.05.

In previous research, we have gained some understanding of *Trem2*. Additionally, we conducted detailed analysis of P2Y12 immunofluorescence staining on spinal cord sections from *Trem2* ^*−/−*^ and Wt mice. Fluorescent images were converted into 8‐bit binary pictures for better visualisation and then into outline pictures for quantitative analysis (Figure 2a). Fractal and lacunarity (FracLac) analysis from ImageJ were applied for quantitative analysis. Microglia from *Trem2* ^*−/−*^ mice showed decreased fractal dimension (Figure 2b), indicating their reduced morphological complexity. Besides, no significant difference in lacunarity was noticed in microglia between *Trem2* ^*−/−*^ and Wt mice, indicating similar morphological heterogeneity and distribution patterns. Four different morphological phenotypes of microglia, ranging from a highly ramified to an amoeboid phenotype, are identified in both murine and human microglia (Figure 2c). We found that microglia from *Trem2* ^*−/*^ mice had reduced ramified phenotype but increased primed and reactive phenotypes (Figure 2d). Together, these data suggest that, under naïve conditions, microglia from *Trem2* ^*−/−*^ mice developed primed and reactive morphological phenotypes with reduced complexity.

**FIGURE 3:**
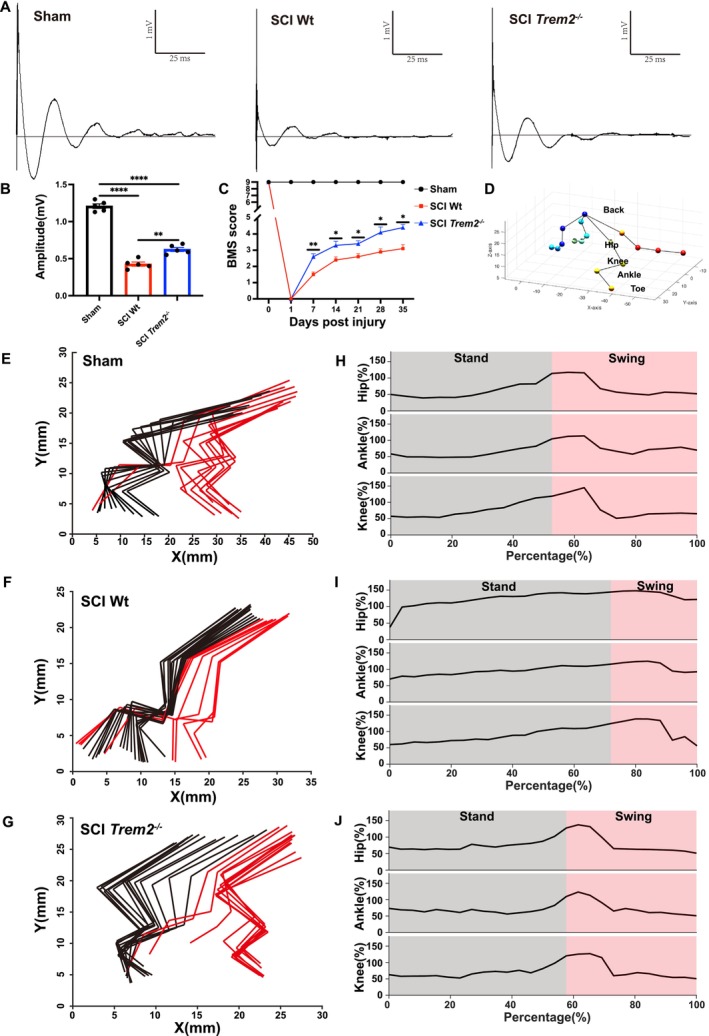
Knocking out *Trem2* in vivo can improve behavioural recovery in spinal cord injury. (A) Representative diagrams of MEP measurements on day 35 following SCI in Sham, SCI wild type and SCI *Trem2* ^*−/−*^ group. (B) Quantitative analysis of the amplitude of the first peak (mV) in (A). *n* = 5. Ordinary one‐way ANOVA. (C) Recovery after SCI assessed by BMS score in Sham, SCI wild type and SCI *Trem2* ^*−/−*^ group; *n* = 5. Ordinary one‐way ANOVA. (D) Diagram of the BehaviorAtlas 3D Gait Analysis model. (E–G) Representative colour‐coded stick views of kinematic hind‐limb movement of sham group, SCI Wt group and SCI *Trem2* ^*−/−*^ group. (H–J) Representative curves of the hip, knee and ankle angles during a one‐step cycle in the sham group, SCI Wt group and SCI *Trem2* ^*−/−*^ group. All data are mean ± SEM; Error bars represent SEMs; *p < 0.05, **p < 0.01, ***p < 0.001, ****p < 0.0001, ns > 0.05.

We reviewed literature and found that morphological changes in microglia may be related to lysosomal functions. Lysosomal membrane permeabilization has emerged as a hot topic in lysosomal research because it significantly affects lysosomal activity. Moreover, improving lysosomal membrane permeabilization is considered an effective method for treating spinal cord injuries. We further attempted to analyse the relationship between *Trem2* and genes associated with lysosomal membrane permeabilization. We discovered that after spinal cord injury, the expression of *Trem2* increases (Figure 3a) and significantly correlates with the expression of genes related to lysosomal membrane permeabilization, such as *Cd68*, *Ctsd*, *Ctsb* and *Ctsz* (Figure 3b–e) [28]. These findings provide an important direction for our subsequent research.

### 3.3 TREM*2* Is Primarily Expressed at the injury centre and Boundary Following Spinal Cord Injury, Which Closely Resembles the Distribution of the Lysosomal Marker Lamp1 After Spinal Cord Injury

**FIGURE 4:**
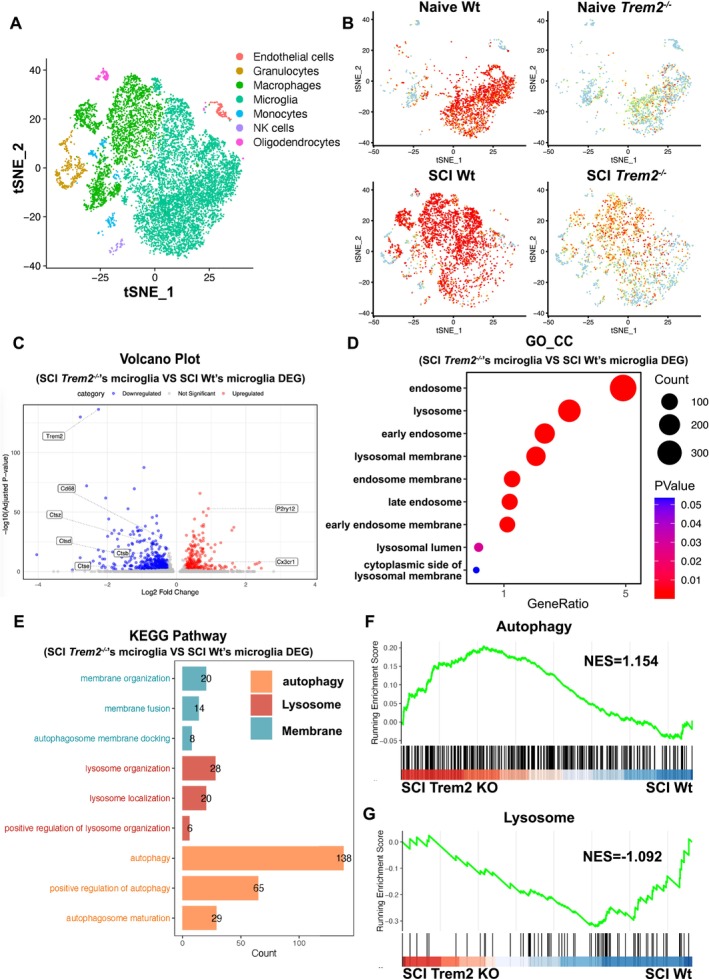
Knocking out *Trem2* in vivo enhances lysosomal activity and autophagy following spinal cord injury. (A, B) t‐SNE plots of cell populations under different genotypes and conditions. Single‐cell RNA‐seq analysis of spinal cord tissue reveals distinct microglial populations across four conditions: Wildtype with naive, wildtype with injury, *Trem2* total knockout with naive and *Trem2* total knockout with injury. The t‐SNE plots illustrate the clustering of major cell types, including microglia, macrophages, monocytes, etc. (C) The volcano plot shows the differential gene expression before and after *Trem2* knockout. Genes significantly upregulated include P2ry12 and Cx3cr1, whilst those significantly downregulated include Trem2, Cd68, Ctsz, Ctsb, Ctsd, Ctse, amongst others. Downregulated genes are marked in blue, whilst upregulated genes are marked in red. (D) Cell component distribution and significance analysis. The dot graph illustrates the quantitative distribution of differentially expressed genes across various cell components before and after *Trem2* knockout. The *x*‐axis represents the gene proportion for each component, whilst the *y*‐axis indicates specific cell components. The size of the circles denotes the number of enriched genes, and the colour indicates the *p*‐value. (E) Pathway functional enrichment analysis of differentially expressed genes. The bar graph illustrates the pathways significantly regulated by differentially expressed genes before and after *Trem2* knockout. Orange represents autophagy‐related pathways, red indicates lysosomal‐related pathways, and blue denotes membrane‐related pathways. The *x*‐axis indicates the number of differentially expressed genes enriched in the regulation of each pathway. (F–G) GSEA for Autophagy and Lysosome Pathways. It shows the enrichment plots comparing the Autophagy (F) and Lysosome (G) pathways between SCI *Trem2* knockout and wildtype mice. The *y*‐axis represents the running enrichment score, and the *x*‐axis shows the rank order of genes based on their differential expression between *Trem2* knockout and wildtype mice.

By analysing single‐cell sequencing data, we demonstrated the expression of *Trem2* before and after spinal cord injury (Figure 2a,b). We quantified the expression levels of *Trem2* at various stages and found that it peaked on the seventh day after the injury (Figure 2c). To further explore the specific cell types expressing TREM2 at the site of spinal cord injury, we performed spinal cord injury surgery on wild‐type mice and collected samples on the seventh day post‐injury. We found that the colocalization of TREM2 with Iba1 was the most pronounced by co‐staining TREM2 with various cell markers, including Iba1 (microglia/macrophage), GFAP (astrocyte), Olig2 (oligodendrocyte) and NeuN (neuron) (Figure 4a–e) [29]. This indicates that TREM2 is primarily expressed in microglia/macrophages. To further validate this finding, we utilised a microglia‐specific fluorescent reporter mouse and observed significant colocalization of TREM2 with TdTomato (a fluorescent marker for microglia) in spinal cord slices taken 7 days post‐injury (Figure 2d). Significantly, the highest expression level of TREM2, as measured by mean fluorescence intensity, is observed at the injury boundary (Figure 2e). To further investigate the distribution of the lysosomal marker Lamp1 after spinal cord injury, we used a microglia‐specific fluorescent reporter mouse and performed immunofluorescence staining of Lamp1 on spinal cord injury sections collected 7 days post‐injury (Figure 2f). The injury boundary was identified by GFAP staining (Figure 4f). Results showed that Lamp1 expression was highest at the boundary of the injury site, which closely resembles the distribution pattern of TREM2 (Figure 2g). This suggests that microglia at the injury boundary may have the highest lysosomal activity, and this activity may be regulated by *Trem2*.

### 3.4 Knockout of *Trem2* Promotes Spinal Cord Injury Recovery In Vivo

**FIGURE 5:**
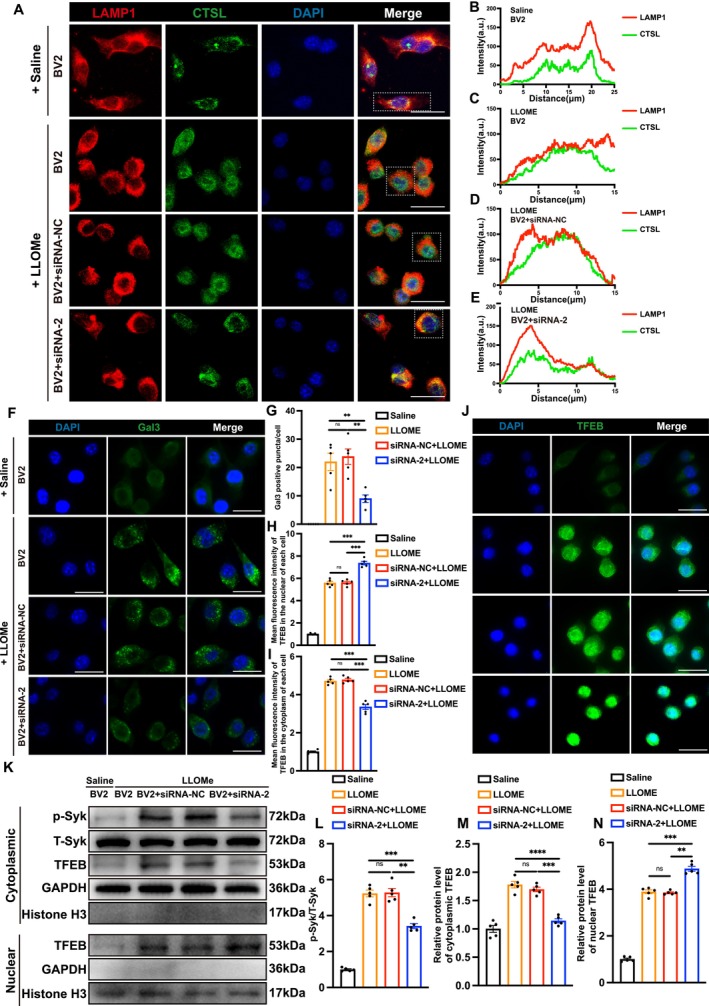
Silencing *Trem2* in vitro promotes autophagy by repairing lysosomal membrane permeabilisation. (A) Immunofluorescence staining of LAMP1 and CTSL in the BV2 + saline, BV2 + LLOMe, BV2 + siRNA‐NC + LLOMe and BV2 + siRNA‐2 + LLOMe groups (scale bar = 20 μm). (B–E) Colocalization analysis of LAMP1 and CTSL in the BV2 + saline, BV2 + LLOMe, BV2 + siRNA‐NC + LLOMe and BV2 + siRNA‐2 + LLOMe groups. (F) Immunofluorescence staining of Gal3 in the BV2 + saline, BV2 + LLOMe, BV2 + siRNA‐NC + LLOMe and BV2 + siRNA‐2 + LLOMe groups (scale bar = 20 μm). (G) Quantification of the number of Gal3 puncta in BV2 cells for each group in (F). *n* = 5. H Mean fluorescence intensity of nuclear TFEB in each cell according to (J). *n* = 5. I Mean fluorescence intensity of cytoplasm TFEB in each cell according to (J). *n* = 5. (J) Immunofluorescence staining of TFEB in the BV2 + saline, BV2 + LLOMe, BV2 + siRNA‐NC + LLOMe and BV2 + siRNA‐2 + LLOMe groups (scale bar = 20 μm). (K) Protein levels of p‐Syk, T‐Syk and TFEB in the cytoplasm and nuclear extracted from BV2 cells of the BV2 + saline, BV2 + LLOMe, BV2 + siRNA‐NC + LLOMe and BV2 + siRNA‐2 + LLOMe groups. (L–N) Quantification of protein levels of p‐Syk, T‐Syk and TFEB in the cytoplasm and nuclear extracted from different groups. *n* = 5. All data are mean ± SEM; Error bars represent SEMs; *p < 0.05, **p < 0.01, ***p < 0.001, ****p < 0.0001, ns > 0.05.

To assess the functional improvements in mice with a *Trem2* knockout following traumatic spinal cord injury, we performed motor evoked potential (MEP) assessments, BMS score and BehaviorAtlas 3D Gait Analysis. First, we bred *Trem2* ^*−/−*^ mice and validated the efficiency of *Trem2* knockout at the DNA, mRNA and protein levels. DNA gel electrophoresis revealed specific fragment sizes of 254 bp in wild‐type mice and 396 bp in *Trem2* ^*−/−*^ mice (Figure 5a). Quantitative PCR (qPCR) results showed that mRNA expression levels in *Trem2* ^*−/−*^ mice were significantly lower than in wild‐type mice (Figure 5b). Immunofluorescence further confirmed the substantial reduction in TREM2 protein levels in *Trem2* ^*−/−*^ mice, demonstrating the high efficiency of the knockout (Figure 5c).

Spinal cord injury surgeries were conducted on wild‐type and *Trem2* ^*−/−*^ mice, with sample collection on day 35 post‐injury. Our results indicated that compared to wild‐type mice, *Trem2* ^*−/−*^ mice exhibited higher motor evoked potentials (Figure 3a,b). Similarly, BMS score indicated that from day seven onwards, *Trem2* ^*−/−*^ mice began to demonstrate superior behavioural performance (Figure 3c). In addition, we performed BehaviorAtlas 3D gait analysis and created a 3D model of the gait (Figure 3d). The kinematic hind‐limb movement diagram of the mice shows that, compared to the SCI Wt group, the SCI *Trem2* ^*−/−*^ group exhibited better gait performance (Figure 3e–g). The representative curves of the hip, knee and ankle angles during a one‐step cycle suggest that, compared to the SCI Wt group, the SCI *Trem2* ^*−/−*^ group has a relatively shorter stance phase and a relatively longer swing phase (Figure 3h,i). This indicates that the gait of the SCI *Trem2* ^*−/−*^ group is closer to that of the sham group, suggesting better recovery from spinal cord injury in the SCI *Trem2* ^*−/−*^ group.

### 3.5 Single‐Cell Sequencing Suggests That Knockout of *Trem2* Promotes Spinal Cord Injury Recovery by Modulating Autophagy and Lysosomal Pathways

We have confirmed that *Trem2* knockout mice achieve better functional recovery after spinal cord injury. To explore the underlying mechanisms by which *Trem2* deletion improves spinal cord injury, we performed spinal cord injury surgeries on both *Trem2* ^*−/−*^ and wild‐type mice and conducted single‐cell RNA sequencing on spinal cord tissues spanning 1 cm around the injury site. The samples were divided into four groups: wild‐type in naive conditions, wild‐type post‐injury, *Trem2* total knockout in naive conditions, and *Trem2* total knockout post‐injury. t‐SNE plots of cell populations under different genotypes and conditions have been generated (Figure 4a). We showed the levels of TREM2 expression in both wild‐type and *Trem2* ^*−/−*^ mice under naive and post‐injury conditions (Figure 4b). Differential gene analysis of wild‐type and *Trem2* ^*−/−*^ mice under post‐injury conditions revealed a volcano plot showing a significant reduction in the expression of TREM2 and lysosomal enzymes Z, D, E and B in *Trem2* ^*−/−*^ mice (Figure 4c). This may be related to improvements in lysosomal membrane permeabilization. Gene Ontology (GO) analysis indicated a significant enhancement of lysosomal and endosomal pathways (including endosomes, lysosomes, early endosomes and lysosomal membranes and more) in *Trem2* ^*−/−*^ mice (Figure 4d). KEGG analysis revealed significant strengthening in the pathways related to autophagy, lysosomes, and membrane‐related processes in *Trem2* ^*−/−*^ mice (Figure 4e). Further, GSEA analysis confirmed that compared to wild‐type, *Trem2* ^*−/−*^ mice exhibit more robust autophagic and lysosomal pathways following spinal cord injury (Figure 4f,g).

### 3.6 Silencing *Trem2* Improves Lysosomal Membrane Permeabilisation by Enhancing TFEB Nuclear Translocation In Vitro

**FIGURE 6:**
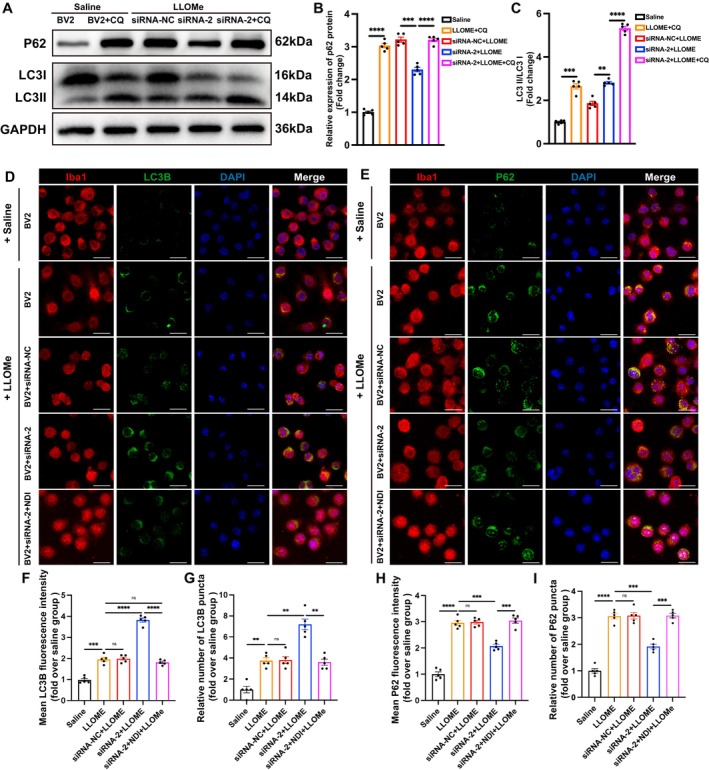
Silencing *Trem2* in vitro promotes autophagy by repairing lysosomal membrane permeabilization. (A) Protein levels of P62 and LC3 from BV2 cells of the BV2 + saline, BV2 + saline + CQ, BV2 + siRNA‐NC + LLOMe, BV2 + siRNA‐2 + LLOMe and BV2 + siRNA‐2 + LLOMe + CQ groups. (B, C) Quantification of protein levels of P62 and LC3 from different groups. *n* = 5. (D) Immunofluorescence staining of LC3B in each group (scale bar = 20 μm). (E) Immunofluorescence staining of P62 in each group (scale bar = 20 μm). (F) Analysis of the mean fluorescence intensity of LC3B in each group (fold change over BV2 + saline group). *n* = 5. Ordinary one‐way ANOVA. (G) Analysis of the relative number of LC3B puncta in each group (fold change over BV2 + saline group). *n* = 5. Ordinary one‐way ANOVA. (H) Analysis of the mean fluorescence intensity of P62 in each group (fold change over BV2 + saline group). *n* = 5. Ordinary one‐way ANOVA. (I) Analysis of the relative number of P62 puncta in each group (fold change over BV2 + saline group). *n* = 5. Ordinary one‐way ANOVA. All data are mean ± SEM; Error bars represent SEMs; *p < 0.05, **p < 0.01, ***p < 0.001, ****p < 0.0001, ns > 0.05.

To validate the results obtained from single‐cell sequencing, we conducted in vitro experiments. First, we constructed three small interfering RNAs (siRNAs) to silence the expression of *Trem2* in BV2 cells. Through Western blot, qPCR and immunofluorescence experiments, we found that the BV2 + siRNA‐2 group exhibited the best silencing effect, showing the lowest mRNA and protein levels (Figure 6a–d). Therefore, in subsequent experiments, we chose siRNA‐2 to silence *Trem2* in BV2 cells. Next, we divided the experiments into four groups: BV2 + saline, BV2 + LLOMe, BV2 + siRNA‐NC + LLOMe and BV2 + siRNA‐2 + LLOMe. L‐Leucyl‐L‐Leucine methyl ester (LLOMe) is a dipeptide condensation product of L‐leucine methyl ester produced by human monocytes or polymorphonuclear leukocytes and is widely used to induce lysosomal membrane permeabilization.

We performed Immunofluorescence on these four groups using LAMP1 (red, marking lysosomes) and CTSL (green, marking the abundant lysosomal protease) (Figure 5a). The results showed that after the addition of LLOMe to BV2 cells, the area of CTSL significantly increased, whilst its colocalization with LAMP1 began to weaken (Figure 5b,c). However, after the introduction of TREM2 small interfering RNA, the area of CTSL started to decrease compared to the control group, and its colocalization with LAMP1 improved (Figure 5d,e). This indicates that the silencing of *Trem2* can improve lysosomal membrane permeability. To provide a more direct quantitative analysis of lysosomal membrane permeabilization, we performed galectin‐3 (gal3) immunofluorescence staining on BV2 cells from each group (Figure 5f). The results showed that the number of gal3 puncta significantly increased in BV2 cells after the addition of LLOMe. However, silencing *Trem2* with small interfering RNA significantly reduced the expression of gal3 puncta (Figure 5g).

The literature indicates that TREM2 is associated with the phosphorylation of Syk, and we propose that this mechanism may be related to the improvement of lysosomal membrane permeabilization upon silencing *Trem2*. First, we performed TFEB immunofluorescence experiments (Figure 5j). The results showed that after silencing *Trem2*, the expression of TFEB increased in the nucleus of BV2 cells, whilst its expression decreased in the cytoplasm (Figure 5h,i). We also conducted Western blot experiments on nuclear and cytoplasmic proteins of BV2 cells (Figure 5k). The results indicated that after silencing *Trem2*, the expression of p‐Syk and TFEB decreased in the cytoplasmic fraction, whilst the expression of TFEB increased in the nuclear fraction (Figure 5l–n). The nuclear translocation of TFEB has been clearly reported in the literature to be associated with lysosomal biogenesis, which can further repair lysosomal membrane permeabilization [30, 31]. Therefore, we propose that silencing *Trem2* can reduce Syk phosphorylation, thereby enhancing its nuclear translocation and ultimately improving lysosomal membrane permeabilization.

### 3.7 Silencing *Trem2* in BV2 Cells Enhances Autophagy In Vitro

We investigated whether silencing *Trem2* could promote autophagic activity by improving lysosomal membrane permeability. The experiments were divided into five groups, followed by Western blot analysis (Note: CQ, chloroquine, is an autophagy flux inhibitor) (Figure 6a). The results revealed that, compared to the Saline group, the expression levels of both LC3II and P62 were significantly elevated in the Saline + CQ group, confirming the effective blockade of autophagy flux by CQ. Moreover, in comparison with the LLOMe + siRNA‐NC group, the LLOMe + siRNA‐2 group exhibited a significant increase in LC3II expression and a marked reduction in P62 expression, indicating that *Trem2* silencing promotes autophagy. Notably, when comparing the LLOMe + siRNA‐2 + CQ group to the LLOMe + siRNA‐2 group, a further increase in LC3II expression was observed, accompanied by a significant rise in P62 expression (Figure 6b,c). These findings suggest that *Trem2* silencing enhances autophagic activity by increasing autophagic flux rather than blocking it.

Next, we divided the in vitro experiments into five groups: Saline, LLOMe, LLOMe + siRNA‐NC, LLOMe + siRNA‐2 and LLOMe + siRNA‐2 + NDI, and performed immunofluorescence staining (Figure 6d,e). The results showed that after the addition of LLOMe, the expression levels of LC3B and P62 in BV2 cells were significantly increased, as evidenced by elevated mean fluorescence intensity and an increased number of LC3 and P62 puncta. We also observed that, compared to the control group, silencing *Trem2* in BV2 cells led to an increase in the mean fluorescence intensity and puncta number of LC3B, whilst the mean fluorescence intensity and puncta number of P62 decreased. However, following the addition of *N*‐dodecylimidazole (NDI) (a lysosomal membrane permeabilization inducer), the mean fluorescence intensity and puncta number of LC3B decreased again, whilst those of P62 increased (Figure 6f–i). These findings suggest that silencing *Trem2* in vitro enhances autophagic activity by restoring lysosomal membrane permeability.

### 3.8 Knocking Out *Trem2* Can Repair Lysosomal Membrane Permeabilisation In Vivo

**FIGURE 7:**
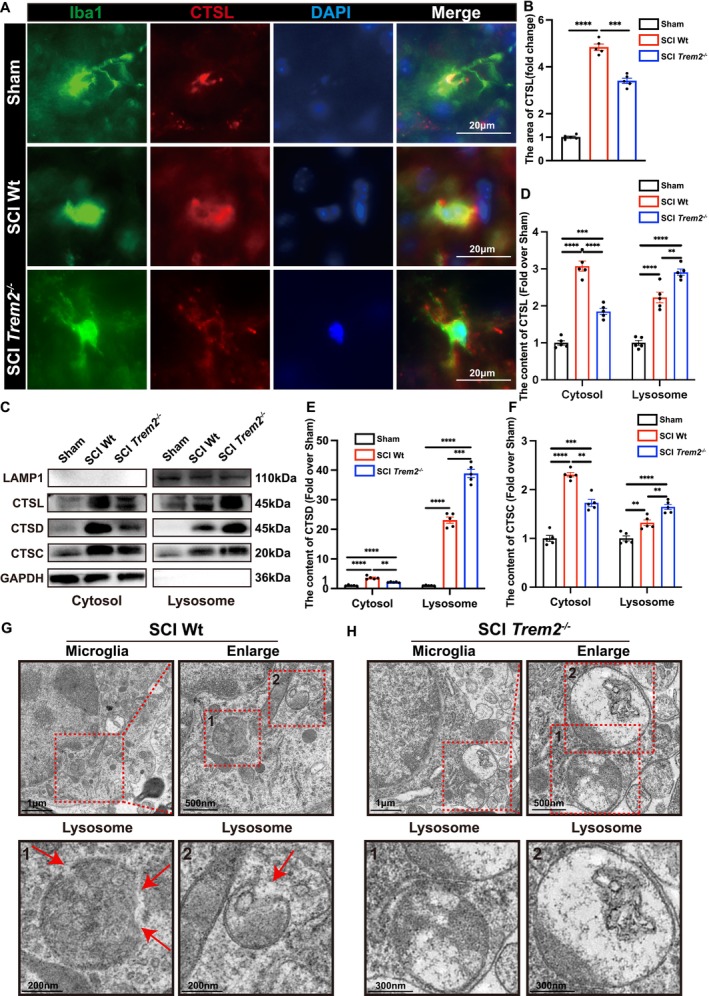
Knocking out *Trem2* in vivo can repair lysosomal membrane permeabilisation after spinal cord injury. (A) Immunofluorescence staining of Iba1 and CTSL in the spinal cord from the Sham, SCI wild type and SCI *Trem2* ^*−/−*^ groups 7 days post‐spinal cord injury (scale bar = 20 μm). (B) Quantification of the diffuse area of CTSL within microglia across different groups. *n* = 5. Ordinary one‐way ANOVA. (C) Protein levels of CTSL, CTSD and CTSC in the cytoplasm and lysosomes extracted from the spinal cords of the Sham, SCI wild type and SCI *Trem2* ^*−/−*^ groups. (D–F). Quantification of the protein levels of (C) CTSL, (E) CTSD, and (F) CTSC in the cytoplasm and lysosomes extracted from the spinal cord. *n* = 5. Ordinary one‐way ANOVA. (G, H).Transmission electron micrographs of lysosomes in microglia at the injury site of mice 7 days post‐spinal cord injury (Red arrows indicate the locations of lysosomal membrane permeabilization). All data are mean ± SEM; Error bars represent SEMs; *p < 0.05, **p < 0.01, ***p < 0.001, ****p < 0.0001, ns > 0.05.

To investigate whether *Trem2* knockout could repair lysosomal membrane permeability after spinal cord injury, I conducted spinal cord injury surgeries on sham‐operated, *Trem2* ^*−/−*^, and wild‐type mouse groups. Seven days post‐injury, we performed immunofluorescence staining on the spinal cord tissues using markers Iba1 (green, indicating microglia) and CTSL (red, indicating cathepsin L) (Figure 7a). The staining results showed that compared to wild‐type mice, the *Trem2* ^*−/−*^ mice had a more concentrated distribution of cathepsin L within microglia, with a significantly reduced area of dispersion (Figure 7b). These findings suggest that *Trem2* knockout may effectively repair lysosomal membrane permeability following spinal cord injury. We prepared lysosome‐enriched fractions through subcellular fractionation of spinal cord tissue, thereby separating the animal tissue proteins into cytosolic and lysosomal components. Subsequently, we detected LAMP1, CTSL, CTSD and CTSC using Western blot analysis (Figure 7c). We found that in the cytosolic components, the expression levels of CTSL, CTSD and CTSC were lower in *Trem2* ^*−/−*^ mice compared to wild‐type mice. However, in the lysosomal components, the expression of these proteins was higher in *Trem2* ^*−/−*^ mice relative to wild‐type mice (Figure 7d–f). In addition, we measured the activity of CTSD in the total spinal cord tissue, lysosomal fractions and cytoplasmic fractions using ELISA. The results showed that, compared to the control group, CTSD activity was significantly reduced in the cytoplasmic fractions and significantly increased in the lysosomal fractions in Trem2 knockout mice, whilst the total CTSD activity showed no significant difference (Figure 7a–c). These findings indicate that Trem2 knockout does not reduce CTSD secretion but rather decreases CTSD leakage into the cytoplasm by reducing lysosomal membrane permeability. To observe the effects of *Trem2* knockout more directly on lysosomal membrane permeability in microglia, we examined spinal cord tissues 7 days post‐injury using electron microscopy. As shown in the figure, compared to *Trem2* ^*−/−*^ mice, wild‐type mice had smaller lysosomes and more obvious lysosomal membrane permeabilization (Figure 7g,h). This finding suggests that knocking out *Trem2* helps improve lysosomal membrane permeability.

### 3.9 Knocking Out *Trem2* In Vivo Promotes Autophagy by Repairing Lysosomal Membrane Permeabilisation in Spinal Cord Injury

**FIGURE 8:**
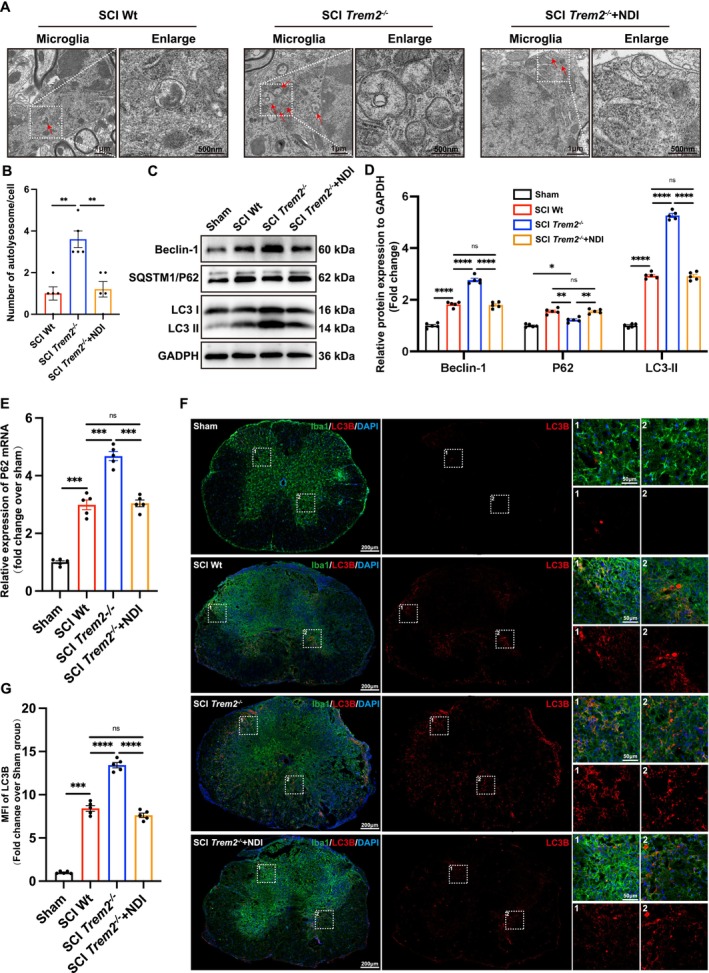
Knocking out *Trem2* in vivo promotes autophagy by repairing lysosomal membrane permeabilisation in spinal cord injury. (A) Transmission electron micrograph of autolysosomes in microglia at the injury site of mice from different groups, 7 days post‐spinal cord injury (red arrows indicate the locations of autolysosomes). (B) Quantification of the number of autolysosomes in transmission electron micrographs from different groups. *n* = 5. Ordinary one‐way ANOVA. (C) Protein levels of Beclin‐1, P62 and LC3 in spinal cord tissues of Sham, SCI wild type, SCI *Trem2* ^*−/−*^ and SCI *Trem2* ^*−/−*^ + NDI mice. (D) Quantification of protein levels of Beclin‐1, P62, and LC3 in the spinal cord tissues of different groups. *n* = 5. Ordinary one‐way ANOVA. (E) The mRNA levels of the P62 gene in the impaired spinal cord of Sham, SCI wild type, SCI *Trem2* ^*−/−*^ and SCI *Trem2* ^*−/−*^ + NDI groups at 7 days post‐injury (dpi). *n* = 5. Ordinary one‐way ANOVA. (F) Immunofluorescence staining of Iba1 and LC3B in the spinal cord from the Sham, SCI wild type, SCI Trem2^−/−^ and SCI *Trem2* ^*−/−*^ + NDI groups 7 days post‐spinal cord injury. (G) Quantifying the mean fluorescence intensity of LC3B in different groups according to graph F. *n* = 5. Ordinary one‐way ANOVA. All data are mean ± SEM; Error bars represent SEMs; *p < 0.05, **p < 0.01, ***p < 0.001, ****p < 0.0001, ns > 0.05.

Our single‐cell sequencing results suggest that knocking out *Trem2* may improve lysosomal membrane permeability and promote autophagy. To further explore whether *Trem2* knockout enhances autophagic function, we utilised transmission electron microscopy, Western blotting, real‐time quantitative PCR (qPCR) and immunofluorescence techniques. On the seventh day post‐spinal cord injury, we examined spinal cord tissues from wild‐type mice, *Trem2* ^*−/−*^ mice and *Trem2* ^*−/−*^ mice + NDI using transmission electron microscopy. The results showed that *Trem2* ^*−/−*^ mice had a significantly higher number of autophagic lysosomes compared to both wild‐type mice and *Trem2* ^*−/−*^ mice + NDI. This suggests that *Trem2* knockout enhances autophagic function by improving lysosomal membrane permeability (Figure 8a,b). Furthermore, our Western blot results showed that, compared to wild‐type mice and *Trem2* ^*−/−*^ mice + NDI groups, *Trem2* ^*−/−*^ mice exhibited a decrease in the autophagy substrate P62, whilst key autophagy proteins Beclin‐1 and LC3 II were significantly increased after spinal cord injury (Figure 8c,d). qPCR results revealed an increase in P62 mRNA levels in *Trem2* ^*−/−*^ mice, indicating active transcription of P62 (Figure 8e). These results further confirm the role of *Trem2* ^*−/−*^ mice in promoting autophagic function. However, when the lysosomal membrane permeability in *Trem2* ^*−/−*^ mice increased, autophagic activity was not enhanced. Finally, we performed immunofluorescence staining on spinal cord tissues 7 days post‐injury and observed a significant increase in the expression of the autophagy marker LC3B in *Trem2* ^*−/−*^ mice compared to other groups (Figure 8f,g). Collectively, these results suggest that *Trem2* knockout significantly enhances autophagic activity following spinal cord injury, and this enhanced autophagic activity may be achieved through the reduction of lysosomal membrane permeability.

### 3.10 Knockout of *Trem2* Promotes Spinal Cord Injury Repair by Improving Histopathology

**FIGURE 9:**
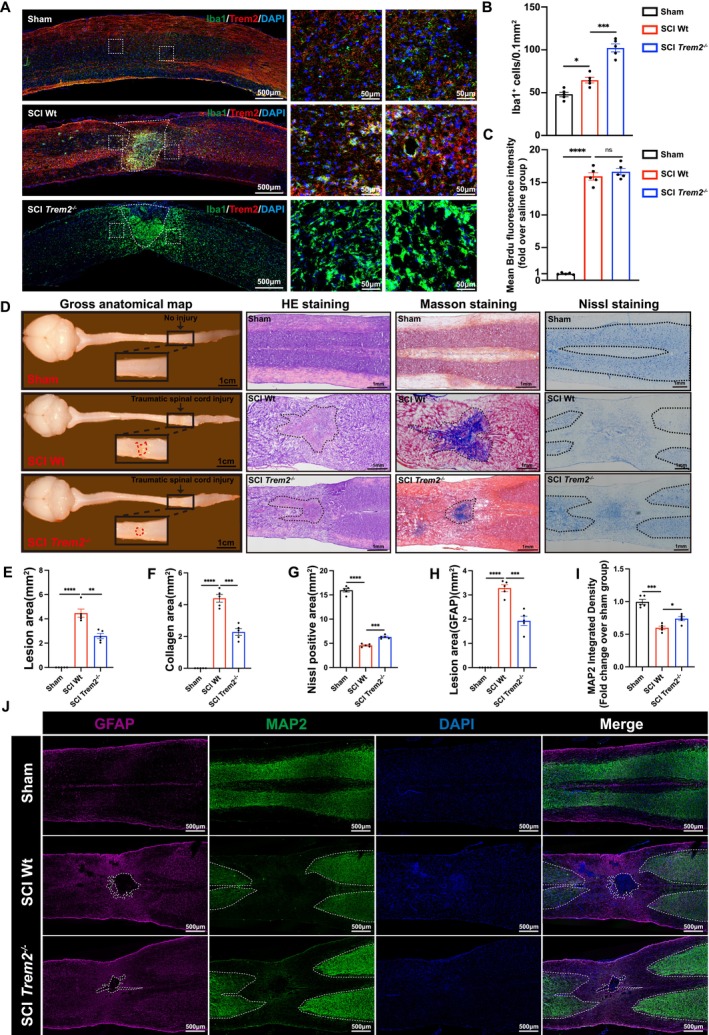
Knocking out *Trem2* in vivo can rescue microglia and promote histological recovery following spinal cord injury. (A) Immunofluorescence staining of Iba1 and TREM2 was performed in the spinal cords of Sham, SCI wild type and SCI *Trem2* ^*−/−*^ mice. (B) Quantification of the density of Iba1^+^ cells (cells/0.1mm^2^). *n* = 5 We selected three regions from the “damage epicentre” and “epicentre boundary” of each sample for quantification, and the average value was calculated for statistical analysis. Ordinary one‐way ANOVA. (C) Analysis of the mean fluorescence intensity of Brdu in each group (fold change over Sham group). *n* = 5. Ordinary one‐way ANOVA. (D) Representative gross anatomical maps, H&E, Masson and Nissl staining images show the lesion core and neuronal region in 35 days after spinal cord injury from Sham, SCI wild type and SCI *Trem2* ^*−/−*^ group. (E) Quantification of lesion areas in each group is based on the results shown in (D). *n* = 5. Ordinary one‐way ANOVA. (F) Quantitative analysis of the collagen area in (D). *n* = 5 Ordinary one‐way ANOVA. (G) Quantification of Nissl positive areas in each group is based on the results shown in (D). *n* = 5. Ordinary one‐way ANOVA. (H) Quantification of lesion areas (GFAP) in each group is based on the results shown in (J). *n* = 5. Ordinary one‐way ANOVA. (I) Quantification of MAP2 integrated density in each group is based on the results shown in (J). *n* = 5. Ordinary one‐way ANOVA. (J) Immunofluorescence staining of GFAP and MAP2 was performed in the spinal cords of Sham, SCI wild type and SCI *Trem2* ^*−/−*^ mice. All data are mean ± SEM; Error bars represent SEMs; *p < 0.05, **p < 0.01, ***p < 0.001, ****p < 0.0001, ns > 0.05.

In the study, we confirmed that the knockout of *Trem2* promotes autophagy by improving lysosomal membrane permeabilization and increasing the production of autophagic lysosomes. Given the increased autophagy in microglia, we further explored whether a greater number of microglia located at the center and periphery of the spinal cord injury could survive. Immunofluorescence results indicated that compared to the Sham group, the SCI Wt group exhibited a higher accumulation of microglia at the injury site (Figure 9a). However, SCI *Trem2* ^*−/−*^ mice displayed a significant increase in both the number and density of microglia at the injury center compared to the SCI Wt group (Figure 9b). Additionally, BrdU staining revealed no significant differences between the SCI Wt and SCI *Trem2* ^*−/−*^ groups (Figure 8a). It suggested that the increase in microglia number and density is attributable to enhanced cell survival due to autophagy rather than an increase in newly generated microglia (Figure 9c).

Further examination of the gross anatomical map of the various groups revealed that the injury area in the SCI *Trem2* ^*−/−*^ group was significantly smaller than that in the SCI Wt group (Figure 9d). HE staining corroborated this finding, showing that the scar area in the SCI *Trem2* ^*−/−*^ group was reduced (Figure 9e). Masson staining revealed that the SCI *Trem2* ^*−/−*^ group exhibited reduced collagen deposition compared to the SCI Wt group (Figure 9f). Nissl staining also indicated a larger positive area for neurons in the SCI *Trem2* ^*−/−*^ group (Figure 9g). To further enhance the reliability of our results, we conducted additional immunofluorescence staining (Figure 9j). The results demonstrated that the area of glial scars, as indicated by GFAP (astrocytes), was significantly smaller in the SCI *Trem2* ^*−/−*^ group compared to the SCI Wt group (Figure 9h). Furthermore, the integrated density of MAP2 (neuronal cell bodies and dendrites) was also higher in the SCI *Trem2* ^*−/−*^ group (Figure 9i). In summary, our findings suggest that the knockout of *Trem2* can rescue more microglia and promote recovery from spinal cord injury by improving histological outcomes.

## 4 Discussion

Prior to spinal cord injury, microglia are one of the major cell types in the spinal cord, and their proportion increases significantly after injury, making them the predominant cell population. This suggests that microglia play a crucial role in the acute repair process following spinal cord injury. Recent literature reports that microglia typically respond rapidly in central nervous system (CNS) diseases, accompanied by significant functional and morphological changes [32, 33]. In SCI, we observed notable changes in microglial density and morphology, which are closely associated with their proximity to the injury epicentre. At the injury centre, microglial numbers are reduced, and their morphology is irregular, likely due to the higher cell death rate in this region. In the peripheral areas of the injury, the number and volume of microglia significantly increase. In areas distant from the injury epicentre, the density and morphology of microglia resemble that of uninjured spinal cord tissue. The morphological changes in microglia are closely linked to their functions, suggesting that microglia' response to spinal cord injury involves not only quantitative changes but also functional regulation.

Triggering Receptor Expressed on Myeloid Cells 2 (TREM2) is considered a major immune signalling hub in neurodegenerative diseases [34, 35]. TREM2 plays a crucial role in regulating microglial function under various pathological conditions. In the existing literature, TREM2 is often reported to exert its effects through the regulation of microglial phagocytosis [36, 37]. For example, in Alzheimer's disease, TREM2 enhances the phagocytosis of β‐amyloid plaques, thereby improving the disease [38, 39]. Through analysis of single‐cell sequencing data in the literature, we identified 11 distinct microglial subpopulations and observed significant changes in the dominant populations from the sham‐operated group to day 7 post‐injury. After spinal cord injury, microglial activity in lysosomal, autophagy and membrane remodelling pathways significantly increased, indicating that these pathways may play a critical role in injury repair. Therefore, in this study, we innovatively link TREM2 with lysosomal membrane permeabilisation and autophagy.

Existing literature has demonstrated that TREM2 can influence the function and morphology of osteoclasts [40, 41]. Since both microglia and osteoclasts are derived from myeloid progenitor cells, TREM2 may have similar effects in microglia. In our study, using *Trem2* ^*−/−*^ mice, we further confirmed that TREM2 can alter the morphology of microglia, indicating that TREM2 may play a role in modulating microglial function after spinal cord injury. Using a microglia‐specific fluorescent reporter mouse, we found that Lamp1 was significantly upregulated at the spinal cord injury boundary, like the distribution pattern of TREM2 after spinal cord injury. This suggests that TREM2 may regulate lysosomal function in terms of spatial localisation. Additionally, we observed a positive correlation between the expression of *Trem2* and genes associated with lysosomal membrane permeabilisation. Lysosomal membrane permeabilisation provides a new explanation for the exacerbation of spinal cord injury due to excessive phagocytosis after injury.

Lysosomal membrane permeabilization (LMP) is a critical type of lysosomal damage, typically triggered by reactive oxygen species (ROS), Fenton reactions within the lysosome, and other cellular stress factors [42]. The characteristic feature of LMP is the increased permeability of the lysosomal membrane, leading to the leakage of lysosomal enzymes (such as cathepsins) into the cytoplasm [11]. These enzymes accumulate in the cytoplasm, whilst the enzymatic activity within the lysosome decreases, resulting in impaired lysosomal function. In recent years, the role of LMP in central nervous system injuries has garnered increasing attention [43, 44]. Studies on spinal cord injury have shown that lysosomal damage leads to the accumulation of neuronal autophagic vesicles and inhibits autophagic flux. However, no effective drugs have yet been identified that can improve spinal cord injury pathology by inhibiting LMP. In this study, we identified a key upstream molecule involved in LMP: TREM2, which presents a potential target for improving LMP.

Through comprehensive experimental analyses, we evaluated functional and histological recovery following spinal cord injury. The results demonstrated that *Trem2* knockout significantly enhanced post‐SCI functional recovery. To elucidate the underlying mechanisms, we performed single‐cell RNA sequencing and GO and KEGG analyses revealed marked upregulation of lysosomal, autophagic and membrane‐related pathways in *Trem2* ^*−/−*^ mice, findings corroborated by transmission electron microscopy (TEM), Western blot and immunofluorescence assays. In vitro experiments further demonstrated that silencing *Trem2* improved lysosomal membrane integrity and augmented autophagic activity by inhibiting Syk phosphorylation to promote TFEB nuclear translocation. The nuclear translocation of TFEB is generally closely associated with its phosphorylation status, where phosphorylated modification leads to TFEB retention in the cytoplasm. Notably, this phosphorylation process may involve regulatory roles of p‐SYK [45]. Whilst recent studies have extensively documented TREM2's downstream signalling via the DAP12‐SYK axis and established TFEB's critical role in lysosomal biogenesis, our work innovatively links TREM2 to TFEB for the first time. By employing lysosomal inhibitors (e.g., chloroquine, CQ), we confirmed that *Trem2* knockout enhances autophagy by increasing autophagic flux rather than blocking it. Additionally, we utilised *N*‐dodecylimidazole (NDI), a lysosomal membrane permeabilization (LMP) inducer composed of a dodecyl alkyl chain and an imidazole ring, to validate the causal relationship between lysosomal membrane integrity and autophagy [24]. Remarkably, NDI administration in *Trem2* ^*−/−*^ mice restored autophagic activity to baseline levels, directly implicating lysosomal membrane repair as the mechanistic driver. Collectively, we conclude that *Trem2* knockout promotes autophagy by reducing SYK phosphorylation to facilitate TFEB‐mediated repair of lysosomal membrane integrity.

It is well known that maintaining appropriate levels of autophagy is crucial for cellular homeostasis and is essential for the function and survival of microglia [46]. Literature reports indicate that the depletion of microglia prior to spinal cord injury severely inhibits recovery [4]. Therefore, restoring and/or enhancing autophagic flux can improve cell survival and accelerate functional recovery after injury. This is consistent with findings from numerous studies on spinal cord injury, which indicate that enhanced autophagy can improve spinal cord injury outcomes. This may be due to increased autophagic activity leading to the accumulation of microglia. The increase in microglial numbers may help form protective scars, effectively limiting the expansion of scar tissue. This process contributes to the histological recovery of spinal cord injury, including a reduction in scar area and an increase in neuronal density. The histological improvements further support the evidence of functional recovery.

## 5 Limitations

Although this study provides an in‐depth investigation into the role of TREM2 in spinal cord injury (SCI) and offers valuable insights into its mechanisms through systemic knockout of *Trem2*, there are still some limitations. First, whilst we demonstrated the reparative effects of *Trem2* knockout on autophagy and lysosomal function in a mouse model of SCI, the pathology and clinical manifestations in mice differ from those in humans with SCI. Therefore, although the results have biological significance, further studies are needed to determine whether these findings can be effectively translated into clinical applications. Second, the absence of *Trem2* inhibitors in our experiments presents an additional challenge for clinical translation in future research.

## 6 Conclusions

This study demonstrates that the knockout of *Trem2* can repair lysosomal membranes, enhance autophagy, and preserve more microglia by reducing SYK phosphorylation to promote TFEB nuclear translocation, thereby improving functional recovery following spinal cord injury. These findings provide theoretical support and novel research directions for improving spinal cord injury through *Trem2* editing, whilst also presenting potential new therapeutic targets for clinical treatment.

## Author Contributions

**Tianlun Zhao, Limin Rong, Senyu Yao:** conceptualization. **Tianlun Zhao, Yu Kang, Haojie Zhang:** methodology. **Tianlun Zhao, Jiawei Di, Haojie Zhang:** investigation. **Tianlun Zhao, Bin Liu:** visualisation. **Tianlun Zhao, Jiawei Di, Limin Rong:** supervision. Tianlun Zhao, Senyu Yao: writing – original draft. **Tianlun Zhao, Senyu Yao, Limin Rong, Haojie Zhang:** writing – review and editing.

## Ethics Statement

This study was approved by the Ethics Committee of Sun Yat‐sen University, Guangzhou, China (Approval number: 2021‐7071; Ethics application number: 2024002263).

## Consent

All authors consent to the publication of this manuscript.

## Conflicts of Interest

The authors declare no conflicts of interest.

## Supporting information


**Data S1.** Supporting Information.

## Data Availability

The data that support the findings of this study are available on request from the corresponding author. The data are not publicly available due to privacy or ethical restrictions.
