# Supplementary material for: TREM2 Impedes Recovery After Spinal Cord Injury by Regulating Microglial Lysosomal Membrane Permeabilization‐Mediated Autophagy
Source: Cell Prolif. 2025 May 4;58(10):e70047. doi: 10.1111/cpr.70047 (PMC12508690; doi:10.1111/cpr.70047)
Supplement: Supplementary file 1 — Data S1. Supporting Information. [file CPR-58-e70047-s001.docx]

Supplementary Materials for

TREM2 impedes recovery after spinal cord injury by regulating microglial lysosomal membrane permeabilization-mediated autophagy

Tianlun Zhao *et al.*

* Limin Rong: ronglm@mail.sysu.edu.cn

**This PDF file includes:**

Supplementary Text

Figs. S1 to S8

Tables 1 to 3

**
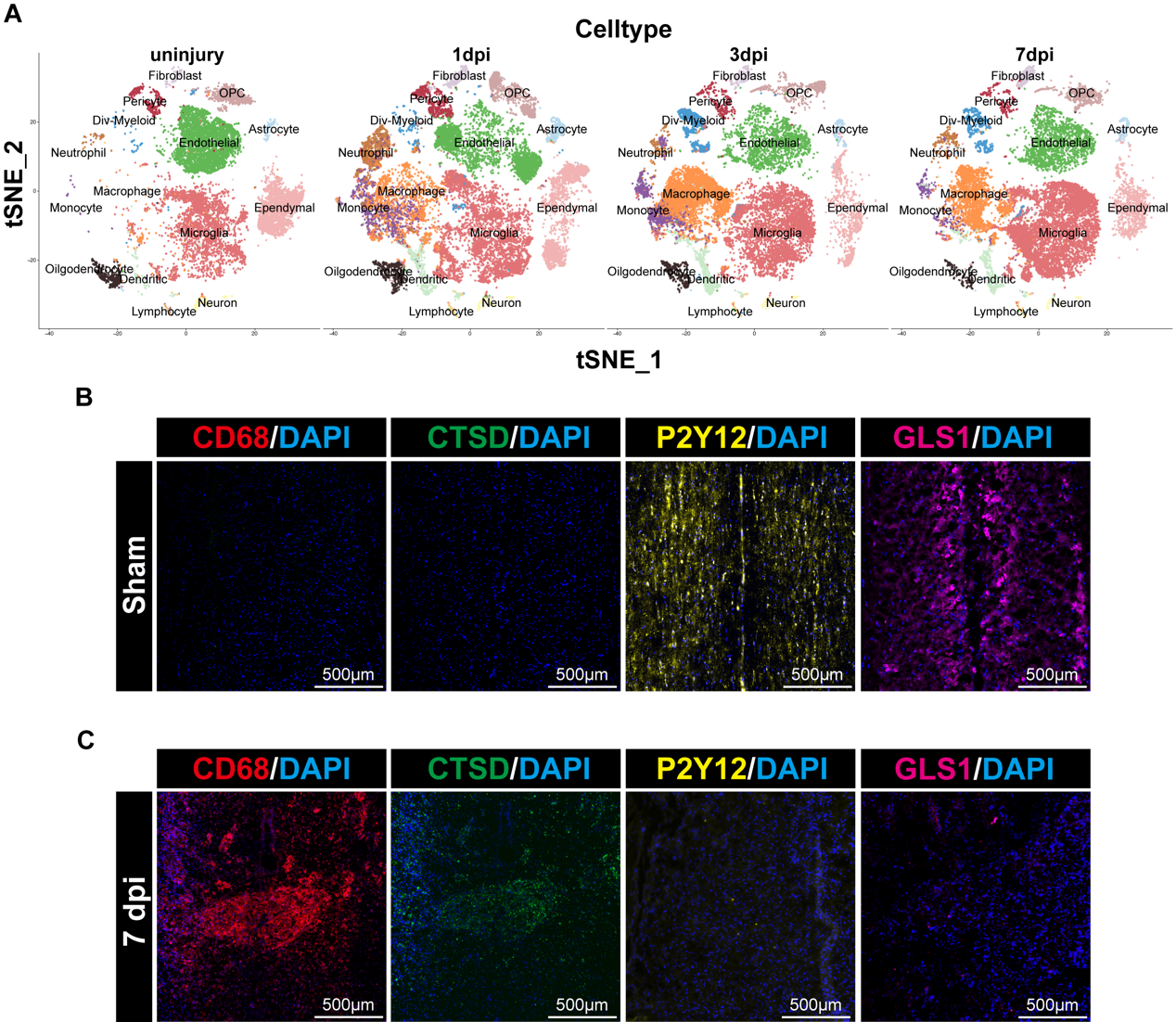
**

**Figs 1: The proportion of microglia increases after spinal cord injury. A** t-SNE plots of cell populations during different time points post-injury. Single-cell RNA-seq analysis of spinal cord tissue reveals distinct cell populations at four time points: uninjured, 1-day post-injury (1 dpi), 3 days post-injury (3 dpi), and 7 days post-injury (7 dpi). The t-SNE plots show clustering of major cell types, including fibroblasts, astrocytes, oligodendrocytes, microglia, macrophages, etc. **B** Representative immunofluorescence images of CD68, CTSD, P2Y12, and GLS1 in spinal cord tissues from the sham group. **C** Representative immunofluorescence images of CD68, CTSD, P2Y12, and GLS1 in spinal cord tissues 7 days after spinal cord injury


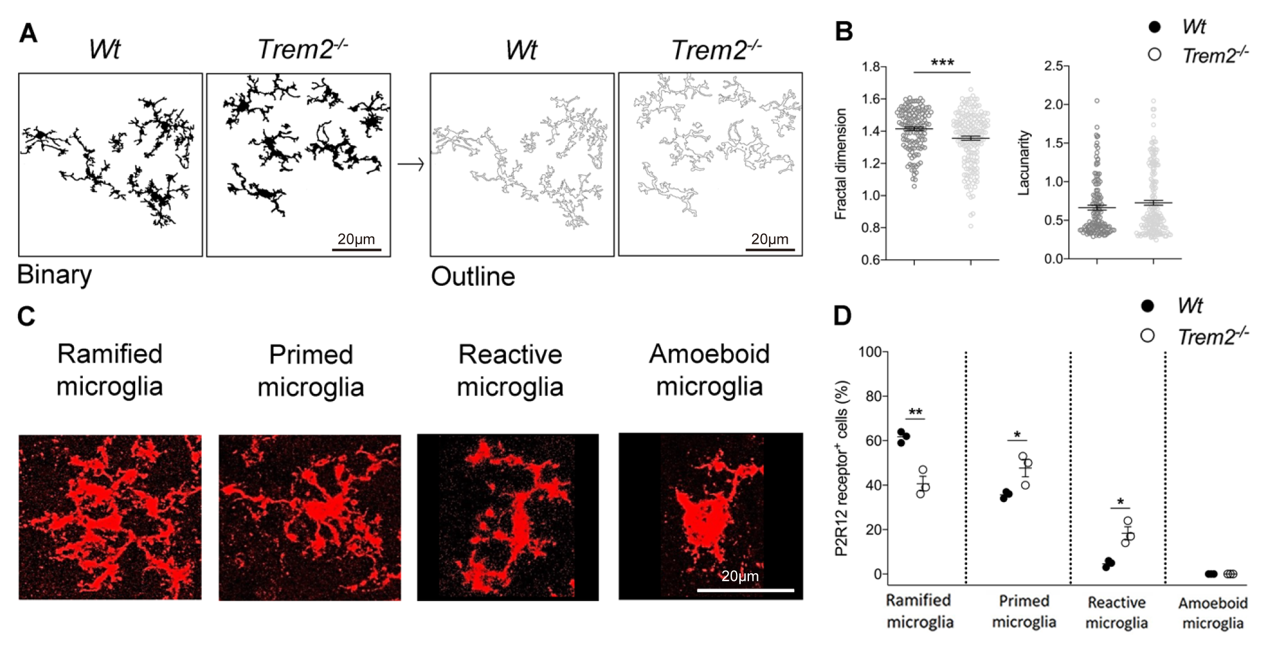


**Figs 2: Microglia from *Trem2^-/-^* mice showed morphological activation patterns in vivo. A** Representative P2Y12 binary and outline pictures of spinal cord sections from *Wt* and *Trem2^-/-^* mice under naïve conditions. **B** Morphological quantification of fractal dimension (left) and lacunarity (right) in microglia from *Wt* and *Trem2^-/-^* mice under naïve conditions. Each dot represents individual microglia cell. n=3 animals/group. Data are mean ± s.e.m. **P<0.01, unpaired two-tailed Student’s t test. **C** Representative P2Y12 immunostaining pictures for different morphological activation patterns of microglia. Scale bar, 20μm. **D** Percentage quantification of different morphological activation patterns in microglia from *Wt* and *Trem2^-/-^* mice under naïve conditions. n=3 animals/group. All data are mean ±SEM; Error bars represent SEMs; *p < 0.05, **p < 0.01, ***p < 0.001, ****p < 0.0001, ns > 0.05.


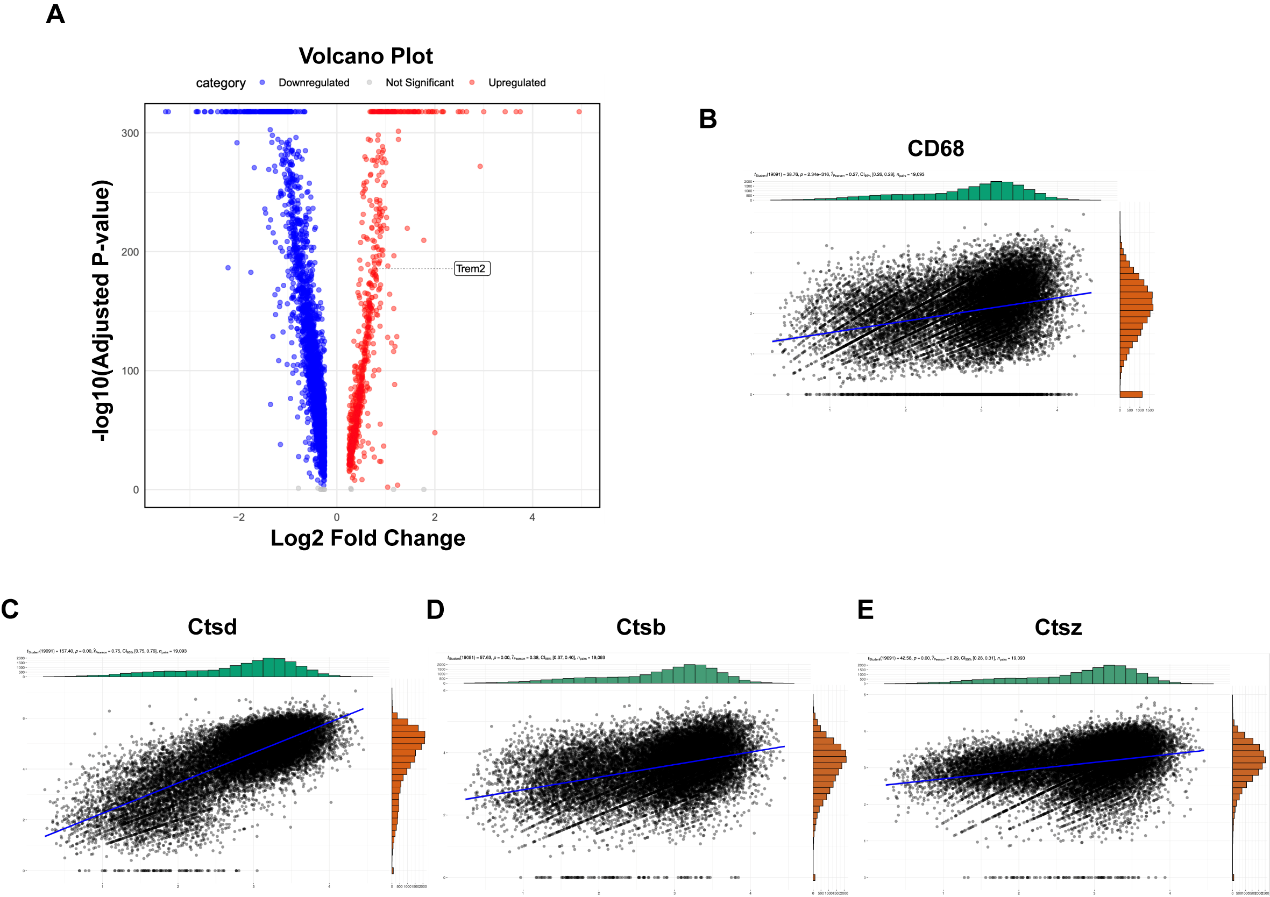


**Figs 3:*Trem2* is highly expressed following spinal cord injury and shows a similar expression trend to CD68, CTSD, CTSB, and CTSZ. A** The volcano plot shows that *Trem2* is highly expressed after spinal cord injury. **B-E** Scatter plot illustrating the correlation between TREM2 and CD68, CTSD, CTSB, and CTSZ expression levels across single cells. Each point represents an individual cell, with density distributions of TREM2 and CD68 expression shown on the x and y margins, respectively. The blue line indicates the linear regression fit, demonstrating a positive correlation between TREM2 and CD68 expression.


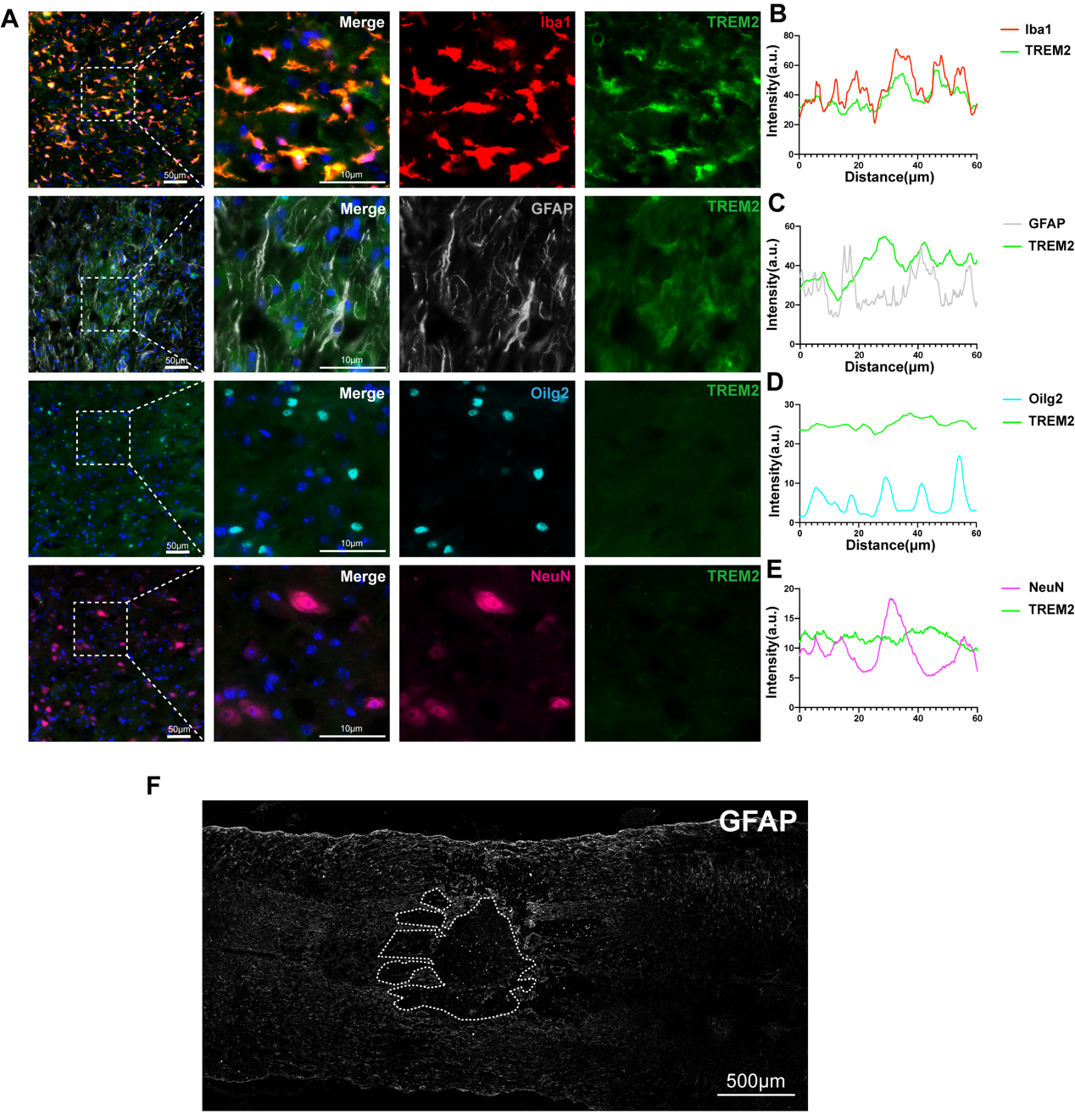


**Figs 4:** **TREM2 is primarily expressed in microglia 7 days after spinal cord injury. A** Expression of TREM2 in macrophages/microglia (Iba1 red), astrocytes (GFAP gray), oligodendrocytes (Olig2 cyan), and neurons (NeuN magenta) in WT mice 7 days following SCI. Scale bar: 50 μm. **B-E** Analysis of the colocalization of TREM2 with macrophages/microglia (Iba1 red), astrocytes (GFAP gray), oligodendrocytes (Olig2 cyan), and neurons (NeuN magenta). **F** Representative immunofluorescence images of GFAP in microglial reporter mice 7 days after spinal cord injury.


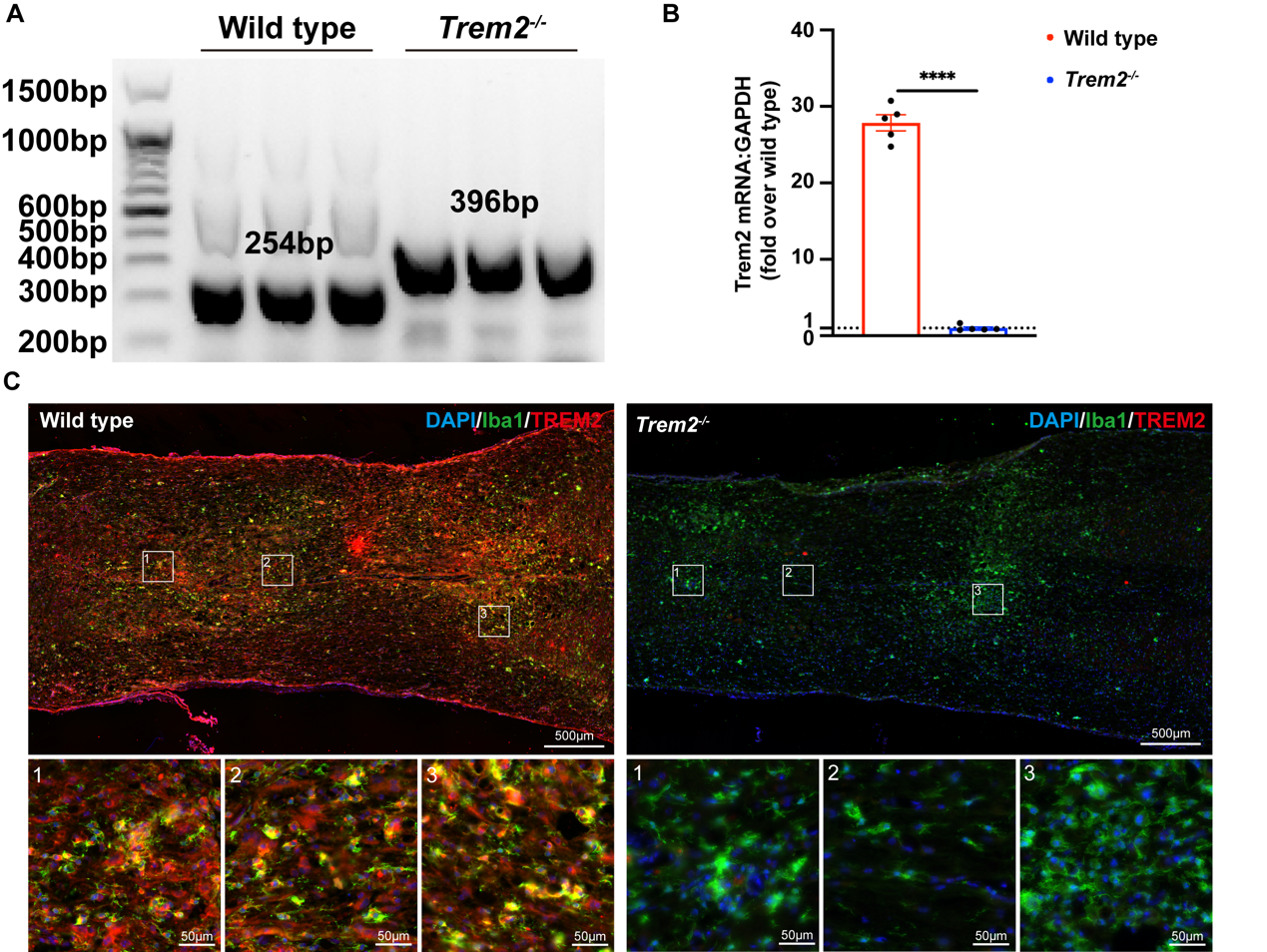


**Figs 5:** **Identification of the knockout efficiency of *Trem2^-/-^* mice. A** The results of DNA gel electrophoresis showed that the wild-type had a band at 254 bp, while the *Trem2^-/-^* showed a band at 396 bp. **B** The relative expression levels of TREM2 mRNA in wild-type and *Trem2^-/-^* mice. ****P<0.0001, unpaired two-tailed Student’s t test.

**C** Immunofluorescence staining of Iba1 and TREM2 in SCI wild-type and SCI *Trem2^-/-^* mice. All data are mean ±SEM; Error bars represent SEMs; *p < 0.05, **p < 0.01, ***p < 0.001, ****p < 0.0001, ns > 0.05.


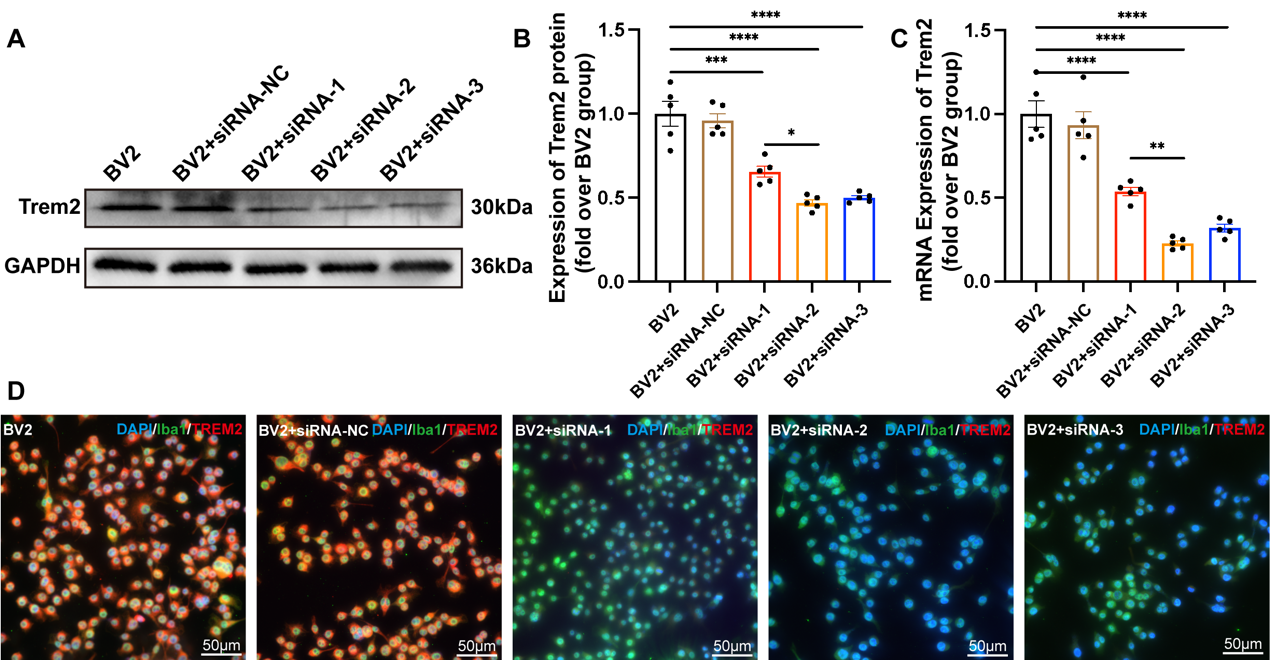


**Figs 6: Identification of the silencing efficiency of *Trem2* in BV2 cells using siRNA. A Protein levels of TREM2 in different groups (BV2 group, BV2+siRNA-NC group, BV2+siRNA-1 group, BV2+siRNA-2 group, and BV2+siRNA-3 group). B Quantification of TREM2 protein levels in different groups. n=5. Ordinary one-way ANOVA. C Quantification of TREM2 mRNA expression in different groups. n=5. Ordinary one-way ANOVA. D Immunofluorescence staining of Iba1 and TREM2 in different groups of BV2 cells. Ordinary one-way ANOVA with two-sided comparisons.** All data are mean ±SEM; Error bars represent SEMs; *p < 0.05, **p < 0.01, ***p < 0.001, ****p < 0.0001, ns > 0.05.


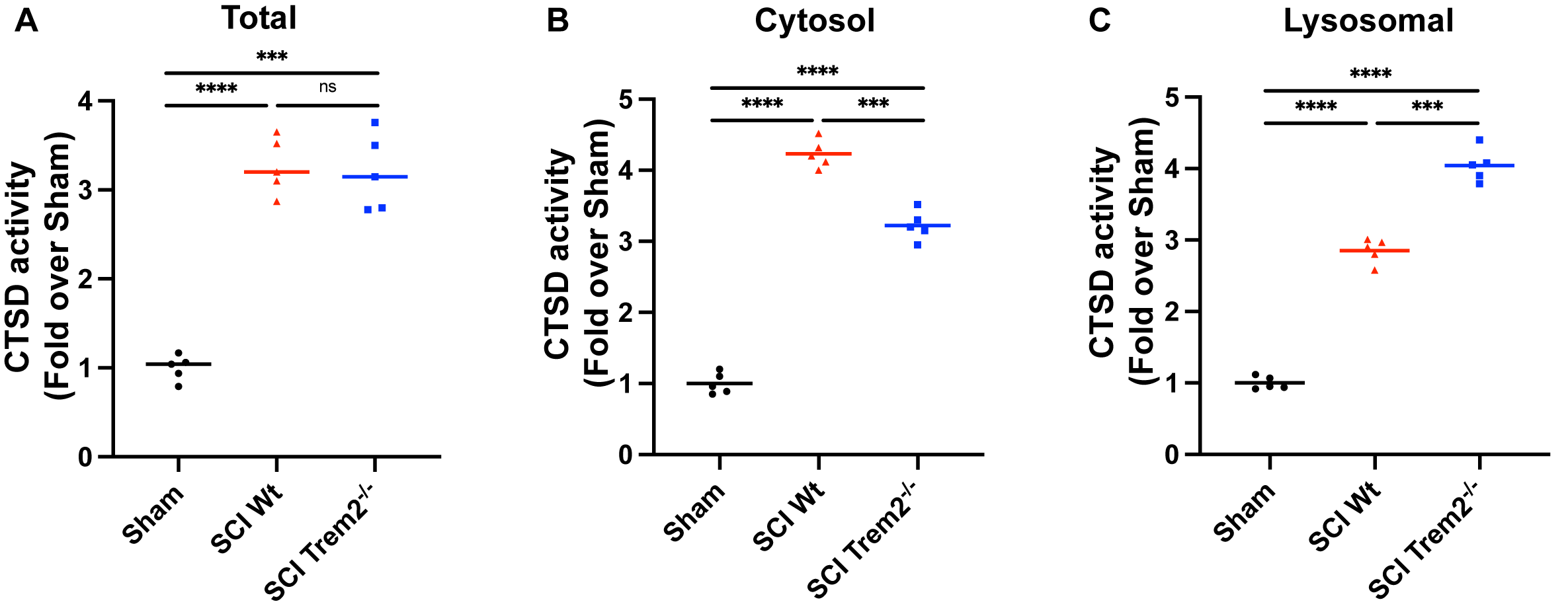


**Figs 7: CTSD activity assay in total protein,** **cytosolic protein, and lysosomal protein fractions from the sham group, wild-type (Wt) group, and Trem2^-/-^ group. A** CTSD activity assay in total protein from the sham group, wild-type (Wt) group, and Trem2^-/-^ group. **B** CTSD activity assay in cytosolic protein from the sham group, wild-type (Wt) group, and Trem2^-/-^ group. **C** CTSD activity assay in lysosomal protein from the sham group, wild-type (Wt) group, and Trem2^-/-^ group.


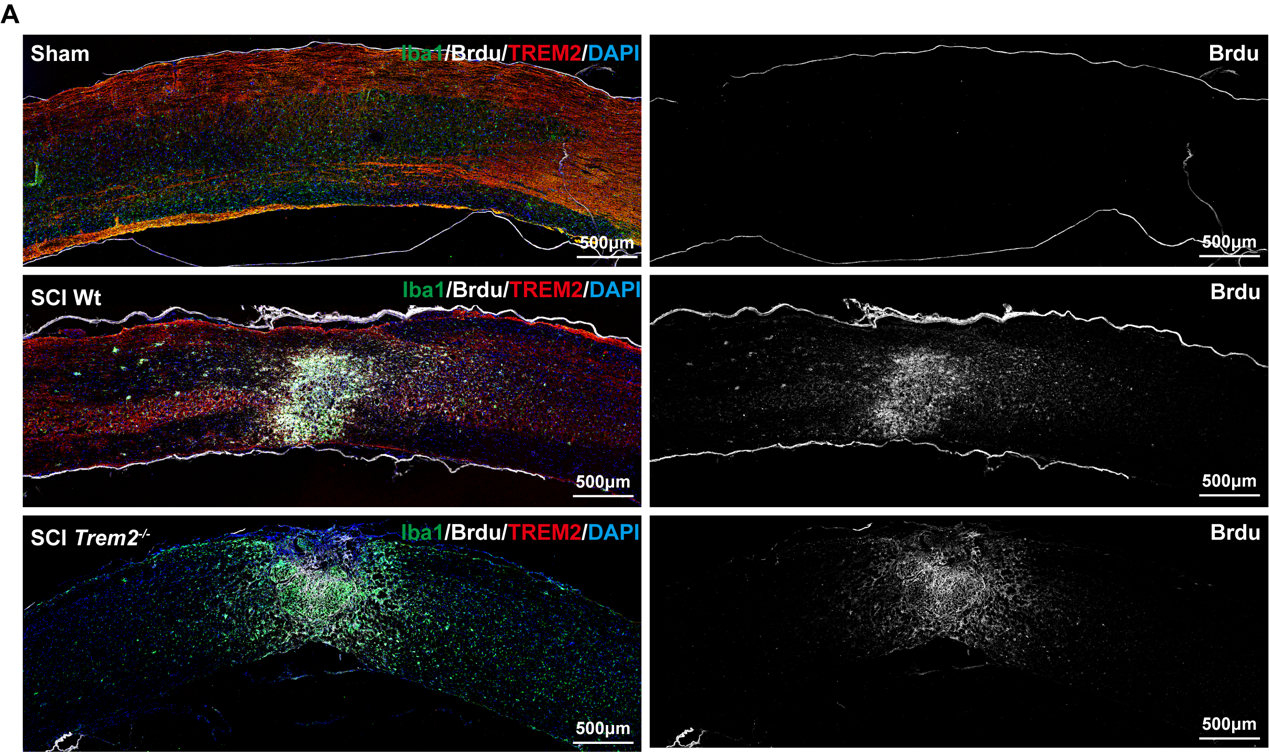


**Figs 8:** **There was no difference in BrdU expression between the SCI wild type and SCI *Trem2^-/-^* groups, but the expression levels were higher compared to the Sham group. A** Immunofluorescence staining of Iba1 and BrdU in different groups.

| Supplementary Table 1: The sequences of protocal primers | | | |
| --- | --- | --- | --- |
| Strains | Primer | Sequence 5'-3' | Primer type |
| **B6.129P2(Cg)-Cx3cr1^tm2.1(cre/ERT2)Litt^/WganJ** | 12266 | AAGACTCACGTGGACCTGCT | Common |
|  | 14314 | CGGTTATTCAACTTGCACCA | Mutant Reverse |
|  | 16221 | AGGATGTTGACTTCCGAGTTG | Wild type Reverse |
| **B6.Cg-Gt(ROSA)26Sor^tm14(CAG-tdTomato)Hze^/J** | olMR9020 | AAGGGAGCTGCAGTGGAGTA | Wild type Forward |
|  | olMR9021 | CCGAAAATCTGTGGGAAGTC | Wild type Reverse |
|  | olMR9103 | GGCATTAAAGCAGCGTATCC | Mutant Reverse |
|  | olMR9105 | CTGTTCCTGTACGGCATGG | Mutant Forward |
| **C57BL/6J-Trem2**^em2Adiuj^/**J** | 28958 | AGTGCTTCAAGGCGTCATAAGT | Wild type Reverse |
|  | 28957 | TCAGGGAGTCAGTCATTAACA | Common |
|  | 28959 | CAATAAGACCTGGCACAACGA | Mutant Reverse |

| Supplementary Table 2: Antibodies resources | | |
| --- | --- | --- |
| Antibodies | Source | Identifier |
| Rat monoclonal anti-TREM2 | R&D System | Cat#MAB17291 |
| Chicken polyclonal anti-GFAP | Abcam | Cat#ab4674 |
| Rabbit ab178846 anti-Iba1 | Abcam | Cat#ab178846 |
| Rabbit polyclonal anti-NeuN | Millipore | Cat#ABN78 |
| Rabbit monoclonal anti-P2Y12 | Invitrogen | Cat#2429941 |
| Rabbit monoclonal anti-GLS1 | CST | Cat#49363 |
| Rabbit monoclonal anti-Oilg2 | Abcam | Cat#ab109186 |
| Rabbit polyclonal anti-LC3 | Proteintech | Cat#14600-1-AP |
| Rabbit polyclonal Anti-LC3B | Abcam | Cat#ab63817 |
| Rabbit polyclonal Anti-p62/SQSTM1 | Abmart | Cat#T55546 |
| RAT monoclonal anti-LAMP1 | Abcam | Cat#ab25245 |
| Mouse monoclonal anti-cathepsin D | Santa cruz | Cat#sc-377299 |
| Mouse monoclonal anti-cathepsin L | Santa cruz | Cat#sc-390367 |
| Mouse monoclonal anti-cathepsin C | Santa cruz | Cat#sc-74590 |
| Rabbit monoclonal anti-Beclin-1 | Abcam | Cat#ab207612 |
| Rabbit polyclonal anti-Histone H3 | Proteintech | Cat#17168-1-AP |
| Rabbit polyclonal anti-Phospho-SYK | Abmart | Cat#TA3315 |
| Rabbit polyclonal anti-TFEB | Abclonal | Cat#A7311 |
| Rabbit monoclonal anti-SYK | Proteintech | Cat#66721-1-AP |
| Goat anti-mouse IgG, Alexa Fluor 488 | Invitrogen | Cat#A-11001 |
| Goat anti-rabbit IgG, Alexa Fluor 488 | Invitrogen | Cat#A-11008 |
| Goat anti-mouse IgG, Alexa Fluor 594 | Invitrogen | Cat#A-11005 |
| Goat anti-rabbit IgG, Alexa Fluor 594 | Invitrogen | Cat#A-11012 |
| Goat anti-chicken IgY, Alexa Fluor 488 | Invitrogen | Cat#A-32931 |
| Goat anti-rat Ig(H&L), Alexa Fluor 647 | Abcam | Cat#ab150167 |
| Goat anti-chicken IgY, Alexa Fluor 680 | Invitrogen | Cat#A-32934 |

| Supplementary Table 3: The sequences of primers | | |
| --- | --- | --- |
| The primer sequences for qPCR | | |
| (Mouse) | sense 5'-3' | antisense 5'-3' |
| Sqstm1/P62 | GATAGCCTTGGAGTCGGT | AAATGTGTCCAGTCATCGTC |
| Trem2 | CAGCCCTGTCCCAAGCCCTCAAC | CTCCTCACCCAGCTGCCGACACC |
| GAPDH | CGGTGCTGAGTATGTCGTGGAGT | CGTGGTTCACACCCATCACAAA |
